# Synthesis of A New Class of Pyridazin-3-one and 2-Amino-5-arylazopyridine Derivatives and Their Utility in the Synthesis of Fused Azines

**DOI:** 10.3390/molecules19022637

**Published:** 2014-02-24

**Authors:** Hamada Mohamed Ibrahim, Haider Behbehani

**Affiliations:** 1 Chemistry Department, Faculty of Science, Kuwait University, P.O. Box 5969, Safat 13060, Kuwait; E-Mail: hamadaaldeb@yahoo.com; 2 Chemistry Department, Faculty of Science, Fayoum University, El-Fayoum 63514, A. R., Egypt

**Keywords:** pyridazin-3-one, 2-amino-5-arylazopyridine, azolo[1,5-*a*]pyrimidine, DMF-DMA, cyanoacetic acid, 1,8-naphthyridine

## Abstract

A general route for the synthesis of a novel class of pyridazin-3-one derivatives **3** by the reaction in acetic anhydride between 3-oxo-2-arylhydrazonopropanals **1** and some active methylene compounds like *p*-nitrophenylacetic acid and cyanoacetic acid was established. Under these conditions the pyridazin-3-one derivatives **3** were formed as the sole isolable products in excellent yield. The 6-acetyl-3-oxopyridazine derivative **3l** was reacted with DMF-DMA to afford the corresponding enaminone derivative **4**, which reacts with a variety of aminoazoles to afford the corresponding azolo[1,5-*a*]pyrimidine derivatives **5**–**7**. Also, in order to explore the viability and generality of a recently uncovered reaction between 3-oxo-2-arylhydrazonopropanals and active methylene compounds, a variety of 2-amino-6-aryl-5-arylazo-3-aroylpyridines **16**–**19** were prepared by reacting 3-oxo-2-arylhydrazonopropanals with miscellaneous active methylene compounds like 3-oxo-3-phenylpropionitrile, hetaroylacetonitriles and cyanoacetamides. These 2-aminopyridine derivatives undergo smooth reactions with cyanoacetic acid that led to the formation in high yield of a new class of 1,8-naphthyridine derivatives **24**. The structures of all new substances prepared in this investigation were determined by the different analytical spectroscopic methods, in addition to the X-ray crystallographic analysis.

## 1. Introduction

**Figure 1:**
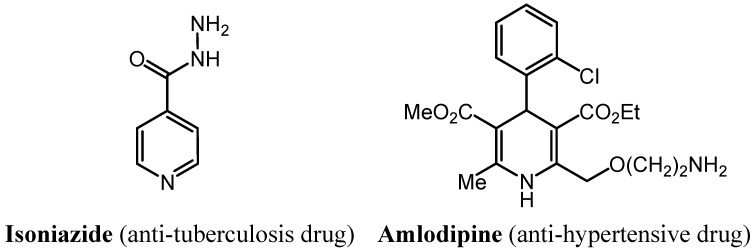
Drugs containing the pyridine motif.

Nitrogen-containing heterocyclic compounds have a diverse range of biological and pharmacological properties [1,2,3]. Pyridazine and pyridine derivatives are two of the most important heterocycles found in medicinal chemistry as they have an excellent biological activity with a wide range of applications, including antimicrobial [4,5,6], antiinflammatory and analgesic [7,8,9], anti-HIV [10], antiplasmodial [11], antitubercular [3,12], antibacterial [3,13], anticonvulsant [14,15], COX inhibitor [16], antidiabetic [17], antihypertensive [18,19], anticancer effects [20,21,22,23,24], blood platelet aggregation inhibitors [25], antidepressant and anxiolytic [26,27], antioxidant [28], antitumor [29,30] and antifungal activities [31]. For example Isoniazide and Amlodipine are two drugs containing the pyridine motif as anti-tuberculosis and anti-hypertensive respectively (Figure 1). On the basis of the above findings the pyridine and pyridazine moieties are considered privileged structures and, consequently, they have attracted the general and continuing interest of synthetic organic chemists.

## 2. Results and Discussion

**Scheme 1:**
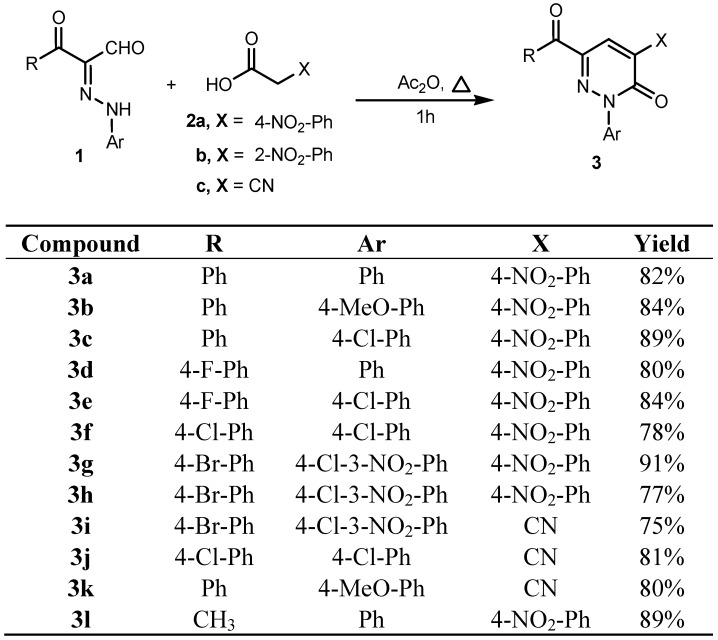
Synthesis of pyridazin-3-one derivatives **3a**–**l**.

**Figure 2:**
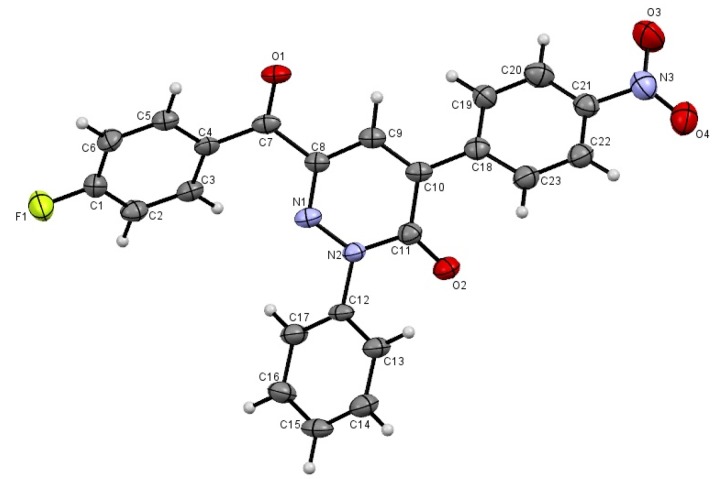
ORTEP plot of the X-ray crystallographic data determined for **3d** [38].

**Figure 3:**
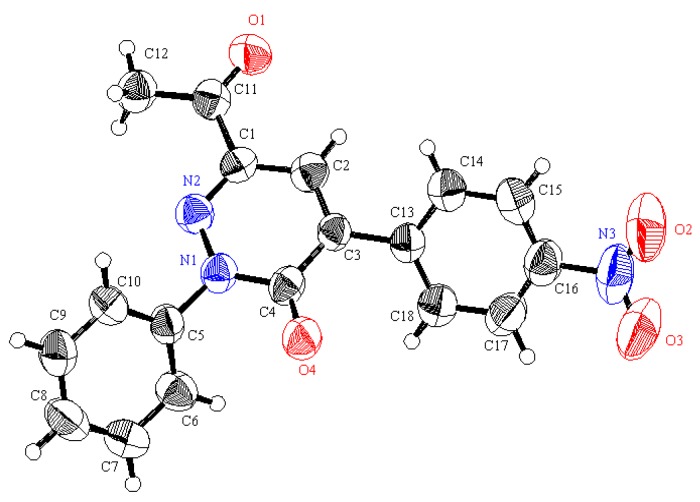
ORTEP plot of the X-ray crystallographic data determined for **3l** [39].

In earlier investigations we developed methods for the efficient synthesis of a variety of polyfunctional azoles, azines and their fused derivatives [32,33,34,35,36]. Recent efforts in our laboratories have led to the design of new and general strategies for the preparation of 2-amino-5-arylazo-nicotinates and pyridazinones [37] that involve reactions of 3-oxo-2-arylhydrazonopropanals **1** with active methylene compounds, including ethyl cyanoacetate, and malononitrile, depending on the effect of the substituent present in the arylazo moiety. Now it was of interest to explore the scope and limitations and generality of the 3-oxo-2-arylhydrazonopropanals **1** as a precursor for the synthesis of some new polyfunctionally substituted pyridazines and pyridines. In order to establish a general route for the synthesis of pyridazin-3-one derivatives **3** as sole products we conducted the reaction between 3-oxo-2-arylhydrazonopropanals **1** and some active methylene compounds, namely *p*-nitrophenylacetic acid (**2a**), *o*-nitrophenylacetic acid (**2b**) and cyanoacetic acid (**2c**) in acetic anhydride. Under these conditions only the pyridazin-3-one derivatives **3** were formed as sole isolable products in excellent yield. The structure of the pyridazin-3-one derivatives **3** was established based on their spectroscopic analyses and X-ray crystallographic analysis (Scheme 1, Figure 2 and Figure 3).

**Scheme 2:**
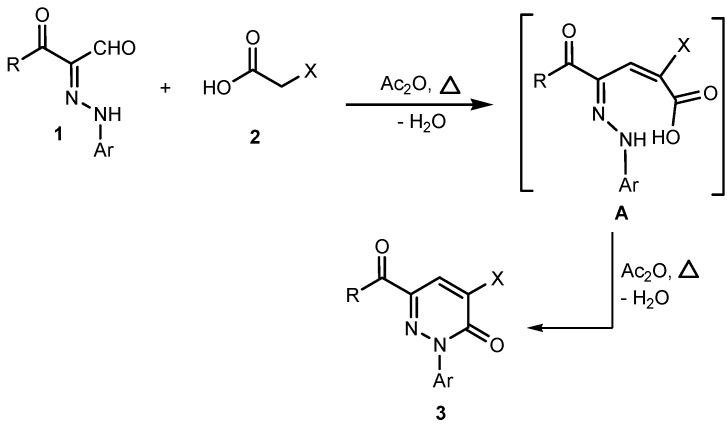
**A** plausible mechanism for the formation of pyridazin-3-ones **3**.

A plausible mechanism for the formation of pyridazin-3-ones **3** (Scheme 2) involves a condensation reaction between the two substrates **1** and **2** to generates the alkylidene intermediate **A**, which then undergoes cyclization via elimination of another water molecule to afford smoothly the pyridazin-3-ones **3**. As illustrated in this mechanism, only two consecutive eliminations of water molecules in the presence of acetic anhydride as a reaction medium were needed to afford only the pyridazin-3-ones **3** in all cases and the formation of the 5-arylazopyridines not observed, due to the absence of ammonium acetate which furnishes the ammonia that plays an essential rule in the formation of the 5-arylazopyridines as described in previous studies [37,40] and also in the forthcoming examples in this study.

**Scheme 3:**
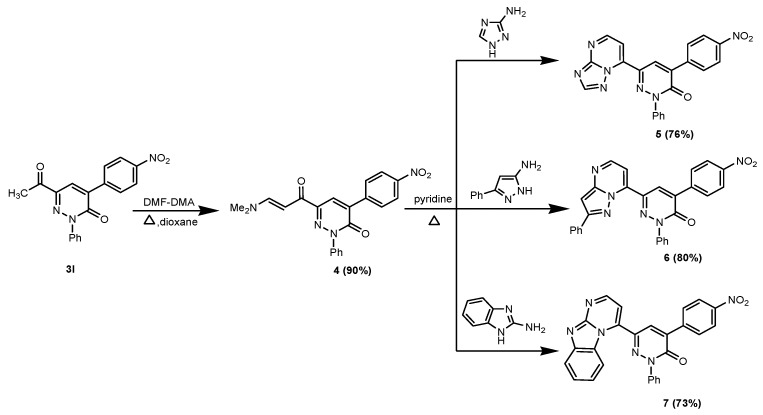
Reactions of the enaminone **4** with heterocyclic amines.

In order to synthesize a new class of enaminone derivatives the 6-acetyl-3-oxopyridazine derivative **3l** was condensed with dimethylformamide dimethylacetal (DMF-DMA) in dioxane to yield the corresponding enaminone **4**, whose ^1^H-NMR spectrum revealed the characteristic two doublet bands for the two olefinic CHs at δ 5.88 and 7.81, respectively, and two signals due to the two methyl groups at δ 2.84 and 3.15, respectively. Moreover MS and HRMS showed its expected M^+^ ion. The foregoing results prompted us to investigate the behaviour of the enaminone **4** towards some *N*-nucleophiles such as heterocyclic amines, as potential precursors of polyfunctionally-substituted fused pyrimidine derivatives for which we expect a broad spectrum of biological activity (Scheme 3).

**Scheme 4:**
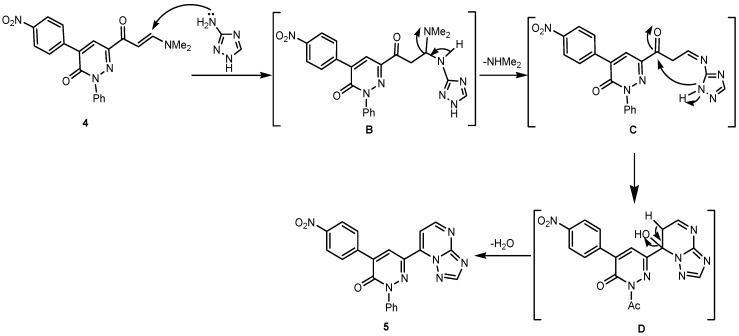
A plausible mechanism for the formation of triazolo[1,5-*a*]pyrimidine derivatives **5**.

Thus the enaminone **4** was reacted with 3-amino-1,2,4-triazole, 3-phenyl-5-aminopyrazole and 2-aminobenzimidazole in refluxing pyridine to afford the corresponding azolo[1,5-*a*]pyrimidine derivatives **5**–**7** that incorporate the pyridazin-3-one moiety. A plausible mechanism for the formation of triazolo[1,5-*a*]pyrimidine derivatives **5** is taken as a representative example to explain the reaction between the enaminone **4** and heterocyclic amines (Scheme 4).

First a Michael-type addition of the exocyclic amino group in the aminotriazole to the α,β-unsaturated moiety in the enaminone **4** yields the corresponding acyclic non-isolable intermediate **B**, which forms the intermediate **C** via elimination of a dimethylamine molecule, and then the intermediate **C** undergoes cyclization through the addition of the NH to CO group to form the intermediate **D**, followed by aromatization via loss of one water molecule to form finally the triazolo[1,5-*a*]pyrimidine derivative **5**.

**Scheme 5:**
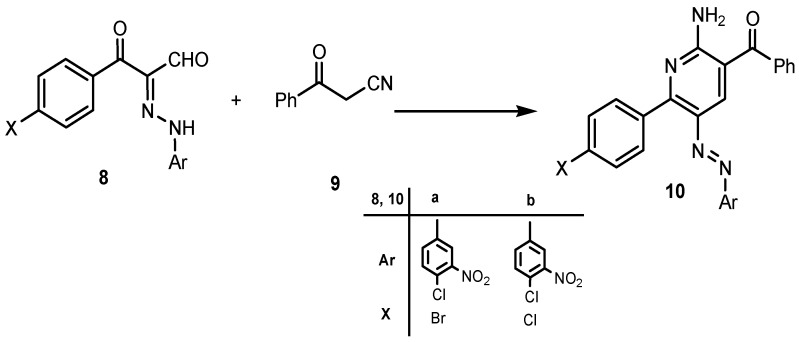
Synthesis of 2-aminopyridines **10** [40].

In a recent study [40] we have also demonstrated that 2-amino-6-aryl-5-arylazo-3-benzoylpyridines **10** were formed as the sole isolable products in the reaction between 3-oxo-3-phenylpropionitrile (**9**) and 3-oxo-2-arylhydrazonopropanals **8a**–**b** containing electron poor arylhydrazone groups as substrates, possessing two electron-withdrawing nitro and Cl groups on the aryl ring of this moiety (Scheme 5).

**Scheme 6:**
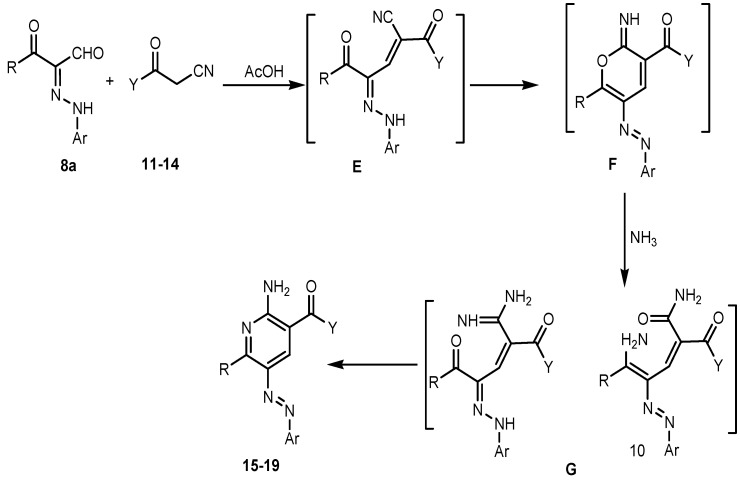
A plausible mechanism for the formation of 2-aminopyridines.

It was therefore of interest to explore the scope, limitations and extend the generality of reaction between 3-oxo-2-arylhydrazonopropanals **8a** with miscellaneous active methylene compounds like 3-oxo-3-phenylpropionitrile, hetaroylacetonitriles and cyanoacetamides to afford polyfunctionally substituted 2-aminopyridines and their utility in the synthesis of 1,8-naphthyridine derivatives. Thus we explored reactions between 3-oxo-2-arylhydrazonopropanal **8a** and cyanoacetylindoles **11**,**12** and different cyanoacetamides **13**,**14**. These processes afford products which were shown to be the respective 2-aminopyridine derivatives **16**–**19** and it is believed that these 2-aminopyridines were formed via the intermediacy of **E**–**G**, as illustrated in the mechanistic pathway presented in (Scheme 6).

**Scheme 7:**
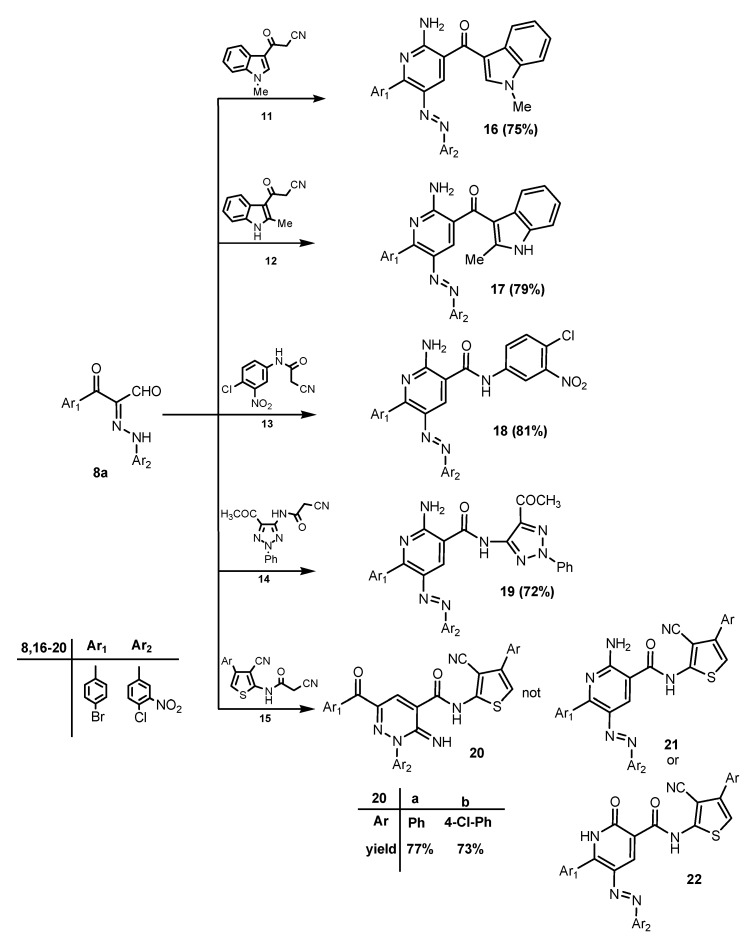
Reaction of arylhydrazonopropanal **8a** with miscellaneous active methylene compounds.

**Figure 4:**
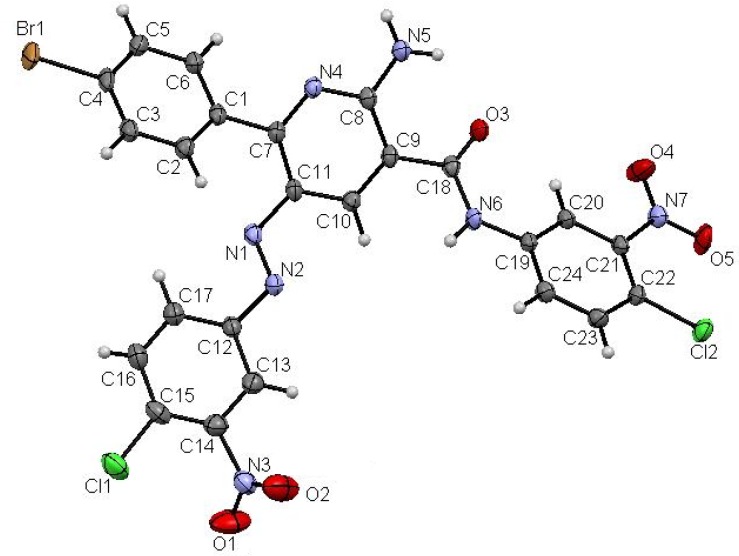
ORTEP plot of the X-ray crystallographic data determined for **18** [42].

In contrast to the observed behaviour of **11**–**14** towards **8a** the thiophene cyanoacetamide **15** behaves differently, affording the pyridazinimine derivatives **20** and not the 2-aminopyridine derivatives **21** or the 2-oxopyridines **22** according to the ^1^H-NMR spectra which showed two signals at δ ≈ 9.80 and 13.3 ppm corresponding to two NH groups, one for the imine NH and the other for the amide NH. Also the ^13^C-NMR spectrum showed a signal at δ ≈ 187.5 corresponding to a true ketone CO and not an amide CO. Till now the factors that make the thiophene cyanoacetamide afford the pyridazine and not pyridine are not clear, but this behaviour may be related to the nature of the thiophene heterocyclic ring and is compatible with an earlier study in which when 3-oxo-2-thiophenehydrazonopropanal reacts with ethyl cyanoacetate, it also affords the pyridazine and not the pyridine system [41]. The structures of these substances were assigned based on their spectroscopic and mass spectrometric properties and X-ray single crystal determination (Scheme 7, Figure 4).

**Scheme 8:**
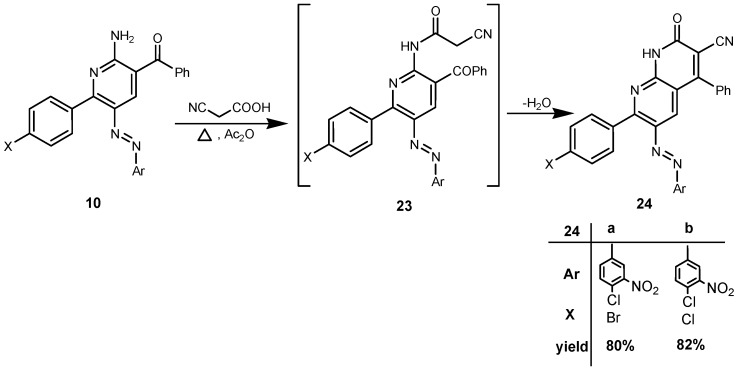
Synthesis of 1,8-naphthyridines **24**.

In order to complete the goal of this study we conducted a reaction between the 2-aminopyridine derivatives **10a**,**b** and cyanoacetic acid in the presence of acetic anhydride to smoothly afford the desired 1,8-naphthyridinecarbonitrile derivatives **24a**,**b** in very good yield. The reaction proceeds most likely via the intermediacy of **23** (Scheme 8).

## 3. Experimental

### 3.1. General

Melting points were recorded and are reported uncorrected. The IR spectra were recorded using KBr pellets on a JASCO FTIR-6300 FT-IR spectrophotometer (Mary’s Court, Easton, MD, USA). ^1^H-NMR (400 MHz) or (600 MHz) and ^13^C-NMR (100 MHz) or (150 MHz) spectra were recorded at 25 °C using CDCl_3_ or DMSO-*d_6_* solutions with TMS as an internal standard on a Bruker DPX 400 or 600 super-conducting NMR spectrometer (Rheinstetten, Germany). Chemical shifts are reported in ppm. Low-resolution electron impact mass spectra [MS (EI)] and high-resolution electron impact mass spectra [HRMS (EI)] were measured using a high resolution GC-MS (DFS) thermo spectrometer at 70.1 eV using magnetic sector mass analyzer (Bremen, Germany). Microanalyses were performed on Elementar-Vario Micro cube Analyzer (Hanau, Germany). Monitoring reactions and determining the homogeneity of the prepared compounds were performed by using thin layer chromatography (TLC) (Sigma-Aldrich). The crystal structures were determined by a Rigaku R-AXIS RAPID diffractometer (Tokyo, Japan) and Bruker X8 Prospector (Madison, WI, USA) and the crystal data collections were made by using Cu-Kα radiation. The data were collected at room temperature. The structure was solved by direct methods and was expanded using Fourier techniques. The non-hydrogen atoms were refined anisotropically. The structure was solved and refined using the Bruker SHELXTL Software Package (Structure solution program-SHELXS-97 and Refinement program-SHELXL-97) [43]. Data were corrected for the absorption effects using the multi-scan method (SADABS). Compounds **10a** and **10b** were prepared according to a literature procedure [40].

### 3.2. Synthesis

#### 3.2.1. General Procedure for the Preparation of Pyridazin-3-one Derivatives **3a**–**l**

Independent mixtures of **1a**–**l** (5 mmol), *p*-nitrophenylacetic acid **2a** or cyanoacetic acid **2b** (5 mmol), in acetic anhydride (10 mL) were stirred at reflux for 1 h. The mixtures were cooled to room temperature. The formed solids were collected by filtration washed by EtOH and recrystallized from the appropriate solvent.

*6-Benzoyl-4-(4-nitrophenyl)-2-phenylpyridazin-3-(2H)-one* (**3a**)*.* Yellow crystals, yield: (1.65 g, 82%), m.p.: 182–183 °C; IR (KBr): *v*/cm^−1^ 1672, 1655 (2CO); ^1^H-NMR (DMSO-*d_6_*): δ =7.50 (t, *J* = 7.6 Hz, 1H, Ar-H), 7.55–7.60 (m, 4H, Ar-H), 7.68–7.72 (m, 3H, Ar-H), 8.093 (d, *J* = 8.0 Hz, 2H, Ar-H), 8.22 (d, *J* = 8.0 Hz, 2H, Ar-H), 8.29 (s, 1H, pyridazine H5), and 8.37–8.39 (m, 2H, Ar-H); ^13^C-NMR (DMSO-*d_6_*): δ = 123.9, 126.6, 128.9, 129.3, 129.4, 129.43, 130.8, 131.1, 133.9, 135.8, 137.9, 140.5, 142.1, 142.9, 148.4, 159.0 and 189.8 ppm (Ar-C and CO); MS (EI): *m/z* (%) 398 ([M + 1]^+^, 28.15), 397 (M^+^, 100); HRMS (EI): *m/z* calcd. for C_23_H_15_N_3_O_4_ (M^+^) 397.1057, found 397.1056. Anal. calcd. for C_23_H_15_N_3_O_4_ (397.39): C, 69.52; H, 3.80; N, 10.57. Found: C, 69.43; H, 3.95; N, 10.66. (([M + 1]^+^ has be changed to ([M + 1]^+^ and please check)

*6-Benzoyl-2-(4-methoxyphenyl)-4-(4-nitrophenyl)pyridazin-3-(2H)-one* (**3b**). Yellow crystals, yield: (1.79 g, 84%), m.p.: 167–168 °C; IR (KBr): *v*/cm^−1^ 1670, 1645 (2CO); ^1^H-NMR (DMSO-*d_6_*): δ = 3.83 (s, 3H, OCH_3_), 7.09 (d, *J* = 8.8 Hz, 2H, Ar-H), 7.58 (t, *J* = 8.0 Hz, 2H, Ar-H), 7.63 (d, *J* = 8.8 Hz, 2H, Ar-H), 7.70 (t, *J* = 8.0 Hz, 1H, Ar-H), 8.08 (d, *J* = 8.0 Hz, 2H, Ar-H), 8.22 (d, *J* = 8.8 Hz, 2H, Ar-H), 8.27 (s, 1H, pyridazine H5) and 8.38 ppm (d, *J* = 8.8 Hz, 2H, Ar-H); ^13^C-NMR (DMSO-*d_6_*): δ = 55.94 (CH_3_), 114.3, 123.8, 127.7, 128.8, 129.2, 130.7, 131.0, 133.8, 134.9, 135.8, 137.6, 140.5, 142.6, 148.3, 159.0, 159.6 and 189.8 ppm (Ar-C and CO); MS (EI): *m/z* (%) 428 ([M + 1]^+^, 34.22), 427 (M^+^, 100); HRMS (EI): *m/z* calcd. for C_24_H_17_N_3_O_5_ (M^+^) 427.1162, found 427.1162. 

*6-Benzoyl-2-(4-chlorophenyl)-4-(4-nitrophenyl)pyridazin-3-(2H)-one* (**3c**). Pale orange crystals, yield: (1.9 g, 89%), m.p.: 188–189 °C; IR (KBr): *v*/cm^−1^ 1672, 1639 (2CO); ^1^H-NMR (DMSO-*d_6_*): δ = 7.50 (t, *J*= 8.0 Hz, 1H, Ar-H), 7.57 (t, *J* = 8.0 Hz, 2H, Ar-H), 7.65 (d, *J* = 8.8 Hz, 2H, Ar-H), 7.71 (d, *J* = 8.0 Hz, 2H, Ar-H), 8.10 (d, *J* = 8.8 Hz, 2H, Ar-H), 8.21 (d, *J* = 8.8 Hz, 2H, Ar-H), 8.27 (s, 1H, pyridazine H5) and 8.38 ppm (d, *J* = 8.8 Hz, 2H, Ar-H); ^13^C-NMR (DMSO-*d_6_*): δ = 123.9, 126.5, 129.0, 129.23, 129.24, 129.3, 130.7, 132.9, 134.4, 137.9, 138.8, 140.3, 141.9, 142.6, 148.3, 158.9 and 188.6 ppm (Ar-C and CO); MS (EI): *m/z* (%) 432 ([M + 1]^+^, 35.87), 431 (M^+^, 100); HRMS (EI): *m/z* calcd. for C_23_H_14_^35^Cl N_3_O_4_ (M^+^) 431.0667, found 431.0669.

*6-(4-Fluorobenzoyl)-4-(4-nitrophenyl)-2-phenylpyridazin-3-(2H)-one* (**3d**). Yellow crystals, yield: (1.7 g, 80%), m.p.: 177–178 °C; IR (KBr): *v*/cm^−1^ 1677, 1655 (2CO); ^1^H-NMR (DMSO-*d_6_*): δ = 7.39 (t, *J* = 8.4 Hz, 2H, Ar-H), 7.47–7.54 (m, 3H, Ar-H), 7.68 (d, *J* = 8.8 Hz, 2H, Ar-H), 8.15–8.18 (m, 4H, Ar-H), 8.24 (s, 1H, pyridazine H5) and 8.34 ppm (d, *J* = 8.8 Hz, 2H, Ar-H); ^13^C-NMR (DMSO-*d_6_*): δ = 115.9, 116.1, 123.8, 126.5, 129.2, 129.3, 130.7, 132.3, 134.03, 134.10, 137.8, 140.3, 142.0, 142.7, 148.3, 158.9, 164.8, 166.4 and 188.2 ppm (Ar-C and CO); MS (EI): *m/z* (%) 416 ([M + 1]^+^, 29.69), 415 (M^+^, 100); HRMS (EI): *m/z* calcd. for C_23_H_14_F N_3_O_4_ (M^+^) 415.0962, found 415.0963. Crystal Data, C_23_H_14_F N_3_O_4_, M = 415.38, triclinic, a = 7.751(3) Å, b = 11.283(4) Å, c = 22.132(8) Å, V = 1873(1) Å^3^, α = 90.895(7)°, β = 97.459(7)°, γ = 102.263(8)°, space group: P-1 (#2), Z = 4, D_calc_ = 1.473 g·cm^−3^, No. of reflection measured 8398, 2 θ _max_ = 54.8°, R1 = 0.0738 [38].

*2-(4-Chlorophenyl)-6-(4-fluorobenzoyl)-4-(4-nitrophenyl)pyridazin-3-(2H)-one* (**3e**). Yellow crystals, yield: (1.9 g, 84%), m.p.: 214–215 °C; IR (KBr): *v*/cm^−1^ 1675, 1643 (2CO); ^1^H-NMR (DMSO-*d_6_*): δ = 7.37–7.40 (m, 2H, Ar-H), 7.60 (d, *J* = 8.4 Hz, 2H, Ar-H), 7.72 (d, *J* = 8.4 Hz, 2H, Ar-H), 8.16–8.18 (m, 4H, Ar-H), 8.23 (s, 1H, pyridazine H5) and 8.33 ppm (d, *J* = 8.4 Hz, 2H, Ar-H); ^13^C-NMR (DMSO-*d_6_*): δ = 115.9, 116.1, 123.8, 128.4, 129.3, 130.7, 132.2, 133.7, 134.05, 134.11, 137.9, 140.2, 140.7, 142.9, 148.3, 158.8, 164.8, 166.5 and 188.1 ppm (Ar-C and CO); MS (EI): *m/z* (%) 450 ([M + 1]^+^, 24.91), 449 (M^+^, 66.15); HRMS (EI): *m/z* calcd. for C_23_H_13_^35^ClFN_3_O_4_ (M^+^) 449.0573, found 449.0573.

*6-(4-Chlorobenzoyl)-2-(4-chlorophenyl)-4-(4-nitrophenyl)pyridazin-3-(2H)-one* (**3f**).Yellow crystals, yield: (1.8 g, 78%), m.p.: 256–257 °C; IR (KBr): *v*/cm^−1^ 1670, 1648 (2CO); ^1^H-NMR (DMSO-*d_6_*): δ = 7.60–7.62 (m, 4H, Ar-H), 7.72 (d, *J* = 8.4 Hz, 2H, Ar-H), 8.07 (d, *J* = 8.4 Hz, 2H, Ar-H), 8.17 (d, *J* = 8.4 Hz, 2H, Ar-H), 8.24 (s, 1H, pyridazine H5) and 8.34 ppm (d, *J* = 8.4 Hz, 2H, Ar-H); ^13^C-NMR (DMSO-*d_6_*): δ = 123.9, 128.3, 129.0, 129.25, 129.30, 130.7, 132.9, 133.7, 134.3, 138.0, 138.88, 140.2, 140.7, 142.7, 148.4, 158.9 and 188.5 ppm (Ar-C and CO); MS (EI): *m/z* (%) 466 ([M + 1]^+^, 40.45), 465 (M^+^, 100); HRMS (EI): *m/z* calcd. for C_23_H_13_^35^Cl_2_N_3_O_4_ (M^+^) 465.0277, found 465.0277.

*6-(4-Bromobenzoyl)-2-(4-chloro-3-nitrophenyl)-4-(4-nitrophenyl)pyridazin-3-(2H)-one* (**3g)**. Yellow crystals, yield: (2.5 g, 91%), m.p.: 236–237 °C; IR (KBr): *v*/cm^−1^ 1676, 1648 (2CO); ^1^H-NMR (DMSO-*d_6_*): δ = 7.80 (d, *J* = 7.6 Hz, 2H, Ar-H), 7.99–8.10 (m, 4H, Ar-H), 8.21 (d, *J* = 7.6 Hz, 2H, Ar-H), 8.29 (s, 1H, pyridazine H5), 8.38 (d, *J* = 7.6 Hz, 2H, Ar-H) and 8.56 ppm (s, 1H, Ar-H); ^13^C-NMR (DMSO-*d_6_*): δ = 123.6, 123.8, 125.7, 128.2, 129.3, 130.7, 131.5, 132.0, 132.7, 132.8, 134.7, 138.5, 139.9, 141.1, 143.4, 147.7, 148.8, 158.9 and 188.4 ppm (Ar-C and CO); MS (EI): *m/z* (%) 556 ([M + 2]^+^, 87.88), 555 ([M + 1]^+^, 41.10), 554 (M^+^, 65.05); HRMS (EI): *m/z* calcd. for C_23_H_12_^79^BrClN_4_O_6_ (M^+^) 553.9623, found 553.9625. 

*6-(4-Bromobenzoyl)-2-(4-chloro-3-nitrophenyl)-4-(2-nitrophenyl)pyridazin-3-(2H)-one* (**3h**). Buff crystals, yield: (2.13 g, 77%), m.p.: 218–219 °C; IR (KBr): *v*/cm^−1^ 1679, 1659 (2CO); ^1^H-NMR (DMSO-*d_6_*): δ = 7.77–7.85 (m, 4H, Ar-H), 7.94–8.01 (m, 3H, Ar-H), 8.07 (d, *J* = 8.8 Hz, 2H, Ar-H), 8.21 (d, *J* = 8.0 Hz, 1H, Ar-H), 8.26 (s, 1H, pyridazine H5) and 8.45 ppm (d, *J* = 2.0 Hz, 1H, Ar-H); ^13^C-NMR (DMSO-*d_6_*): δ = 123.5, 124.9, 125.8, 128.2, 128.4, 128.7, 131.3, 131.8, 132.0, 132.8, 133.0, 133.1, 134.5, 134.9, 140.5, 140.9, 143.5, 147.6, 148.7, 158.3 and 188.4 ppm (Ar-C and CO); MS (EI): *m/z* (%) 556 ([M + 2]^+^, 8.85), 555 ([M + 1]^+^, 3.44), 554 (M^+^, 5.15); HRMS (EI): *m/z* calcd. for C_23_H_12_^79^BrClN_4_O_6_ (M^+^) 553.9623, found 553.9624.

*6-(4-Bromobenzoyl)-2-(4-chloro-3-nitrophenyl)-3-oxo-2,3-dihydropyridazine-4-carbonitrile* (**3i**). Brown crystals, yield: (1.7 g, 75%), m.p.: above 300 °C; IR (KBr): *v*/cm^−1^ 2220 (CN), 1688, 1654 (2CO); ^1^H-NMR (DMSO-*d_6_*): δ = 7.79 (d, *J* = 8.0 Hz, 2H, Ar-H), 7.98 (d, *J* = 8.0 Hz, 2H, Ar-H), 8.01–8.04 (m, 2H, Ar-H), 8.47 (s, 1H, pyridazine H5) and 8.83 ppm (s, 1H, Ar-H); ^13^C-NMR (DMSO-*d_6_*): δ = 113.8, 116.1, 123.6, 126.5, 128.6, 131.5, 132.1, 133.0, 133.1, 134.0, 139.5, 139.9, 142.3, 147.5, 156.5 and 187.5 ppm (Ar-C, CN and CO); MS (EI): *m/z* (%) 460 ([M + 2]^+^, 22.58), 459 ([M + 1]^+^, 7.04), 458 (M^+^, 16.81); HRMS (EI): *m/z* calcd. for C_18_H_8_^79^BrClN_4_O_4_ (M^+^) 457.9411, found 457.9411.

*6-(4-Chlorobenzoyl)-2-(4-chlorophenyl)-3-oxo-2,3-dihydropyridazine-4-carbonitrile* (**3j**). Brown crystals, yield: (1.5 g, 81%), m.p.: above 300 °C; IR (KBr): *v*/cm^−1^ 2218 (CN), 1689, 1662 (2CO); ^1^H-NMR (DMSO-*d_6_*): δ = 7.62–7.70 (m, 4H, Ar-H), 7.96 (d, *J* = 8.4 Hz, 2H, Ar-H), 8.00 (d, *J* = 8.4 Hz, 2H, Ar-H) and 8.77 ppm (s, 1H, pyridazine H5); MS (EI): *m/z* (%) 370 ([M + 1]^+^, 35.22), 369 (M^+^, 59.38); HRMS (EI): *m/z* calcd. for C_18_H_9_Cl_2_N_3_O_2_ (M^+^) 369.0066, found 369.0063.

*6-Benzoyl-2-(4-methoxyphenyl)-3-oxo-2,3-dihydropyridazine-4-carbonitrile* (**3k**). Brown crystals, yield: (1.4 g, 80%), m.p.: 164–165 °C; IR (KBr): *v*/cm^−1^ 2219 (CN), 1695, 1659 (2CO); ^1^H-NMR (DMSO-*d_6_*): δ = 3.85 (s, 3H, O*CH_3_*), 7.07 (d, *J* = 8.8 Hz, 2H, Ar-H), 7.35–7.65 (m, 5H, Ar-H), 8.04 (d, *J* = 8.4 Hz, 2H, Ar-H) and 8.75 ppm (s, 1H, pyridazine H5); MS (EI): *m/z* (%) 332 ([M + 1]^+^, 16.85), 331 (M^+^, 68.11); HRMS (EI): *m/z* calcd. for C_19_H_13_N_3_O_3_ (M^+^) 331.0951, found 331.0949.

*6-Acety-4-(4-nitrophenyl)l-2-phenylpyridazin-3-(2H)-one* (**3l**). Yellow crystals, yield: (1.5 g, 89%), m.p.: 182–183 °C; IR (KBr): *v*/cm^−1^ 1689, 1672 (2CO); ^1^H-NMR (DMSO-*d_6_*): δ = 2.51 (s, 3H, CH_3_), 7.51 (t, *J* = 7.8 Hz, 1H, Ar-H), 7.58 (t, *J* = 7.8 Hz, 2H, Ar-H), 7.70 (d, *J* = 7.8 Hz, 2H, Ar-H), 8.11 (s, 1H, pyridazine H5), 8.14 (d, *J* = 8.4 Hz, 2H, Ar-H) and 8.31 ppm (d, *J* = 8.4 Hz, 2H, Ar-H); ^13^C-NMR (DMSO-*d_6_*): δ = 25.1 (CH_3_), 123.8, 126.4, 126.8, 129.25, 129.30, 130.7, 137.5, 140.4, 142.0, 142.5, 148.3, 159.1 and 195.1 ppm (Ar-C and CO); MS (EI): *m/z* (%) 336 ([M + 1]^+^, 25.08), 335 (M^+^, 100); HRMS (EI): *m/z* calcd. for C_18_H_13_N_3_O_4_ (M^+^) 335.0900, found 335.0901. Crystal Data, C_18_H_13_N_3_O_4_, M = 335.32, monoclinic, a = 25.68(3) Å, b = 3.848(4) Å, c = 32.02(3) Å, V = 3164(5) Å^3^, α = γ = 90°, β = 90.62(2)°, space group: C2/c (#15), Z = 8, D_calc_ = 1.408 g·cm^−3^, No. of reflection measured 2806, 2 θ _max_ = 50.1°, R1 = 0.0818 [39].

*(E)-6-(3-(Dimethylamino)acryloyl)-4-(4-nitrophenyl)l-2-phenylpyridazin-3-(2H)-one* (**4**). Mixture of **3l** (1.68 g, 5 mmol), *N*,*N*-dimethylformamide dimethylacetal (DMF-DMA) (0.6 g, 5 mmol) in dioxane (20 mL) were stirred at reflux for 5 h. The separated solid product obtained on standing at room temperature was collected by filtration, washed by EtOH and recrystallized from dioxane to afford the corresponding enamines **4** as orange crystal. Yellow crystals, yield: (1.70 g, 90%), m.p.: 214–215 °C; IR (KBr): *v*/cm^−1^ 1678, 1644 (2CO); ^1^H-NMR (DMSO-*d_6_*): δ = 2.84 (s, 3H, CH_3_), 3.15 (s, 3H, CH_3_), 5.88 (d, *J* = 12 Hz, 1H, olefinic CH=CH), 7.49 (t, *J* = 7.8 Hz, 1H, Ar-H), 7.56 (t, *J* = 7.8 Hz, 2H, Ar-H), 7.67 (d, *J* = 7.8 Hz, 2H, Ar-H), 7.81 (d, *J* = 12 Hz, 1H, olefinic CH=CH), 8.13 (d, *J* = 8.4 Hz, 2H, Ar-H), 8.15 (s, 1H, pyridazine H5) and 8.30 ppm (d, *J* = 8.4 Hz, 2H, Ar-H); ^13^C-NMR (DMSO-*d_6_*): δ = 37.6 (CH_3_), 45.1 (CH_3_), 88.6, 123.7, 126.6, 127.9, 128.9, 129.30, 130.6, 137.3, 140.9, 142.3, 144.3, 148.1, 155.1, 159.1 and 180.7 ppm (Ar-C and CO); MS (EI): *m/z* (%) 391 ([M + 1]^+^, 19.75), 335 (M^+^, 85.07); HRMS (EI): *m/z* calcd. for C_21_H_18_N_4_O_4_ (M^+^) 390.1322, found 390.1322. Anal. calcd. for C_21_H_18_N_4_O_4_ (390.40): C, 64.61; H, 4.65; N, 14.35. Found C, 64.80; H, 4.77; N, 14.53.

#### 3.2.2. General Procedure for the Synthesis of Azolopyrimidines **5**–**7**

Independent mixtures of **4** (0.78 g, 2 mmol) and the appropriate heteroaromatic amine (2 mmol) in pyridine (20 mL) were stirred at reflux for 24 h. The reaction mixtures were cooled to room temperature and poured into ice cold water then acidified with hydrochloric acid (2 N), forming solids that were collected by filtration and washed with water then MeOH and recrystallized from the appropriate solvent.

*4-(4-Nitrophenyl)-2-phenyl-6-(1,2,4-triazolo[1,5-a]pyrimidin-7-yl)-(2H)-pyridazin-3-one* (**5**). Pall yellow crystals, yield: (0.7 g, 76%), m.p.: 149–150 °C; IR (KBr): *v*/cm^−1^ 1668 (CO); ^1^H-NMR (DMSO-*d_6_*): δ = 7.54 (t, *J* = 8.0 Hz, 1H, Ar-H), 7.61 (t, *J* = 8.0 Hz, 2H, Ar-H), 7.79 (d, *J* = 8.0 Hz, 2H, Ar-H), 7.98 (d, *J* = 5.4 Hz, 1H, pyrimidine H6), 8.25 (d, *J* = 8.0 Hz, 2H, Ar-H), 8.38 (d, *J* = 8.0 Hz, 2H, Ar-H), 8.66 (s, 1H, pyridazine H5), 8.80 (s, 1H, triazole H2) and 9.48 ppm (d, *J* = 5.4 Hz, 1H, pyrimidine H5); MS (EI): *m/z* (%) 461 ([M + 1]^+^, 30.78), 460 (M^+^, 100); HRMS (EI): *m/z* calcd. for C_26_H_16_N_6_O_3_ (M^+^) 460.1278, found 460.1278.

*4-(4-Nitrophenyl)-2-phenyl-6-(2-phenylpyrazolo[1,5-a]pyrimidin-7-yl)-(2H)-pyridazin-3-one* (**6**). Yellow crystals, yield: (0.77 g, 80%), m.p.: 285–286 °C; IR (KBr): *v*/cm^−1^ 1671 (CO); ^1^H-NMR (DMSO-*d_6_*): δ = 7.43–7.45 (m, 2H, Ar-H), 7.49 (d, *J* = 4.8 Hz, 1H, pyrimidine *H6*), 7.51–7.54 (m, 3H, Ar-H), 7.59 (t, *J* = 7.8 Hz, 2H, Ar-H), 7.80 (d, *J* = 7.8 Hz, 2H, Ar-H), 8.05 (d, *J* = 7.8 Hz, 2H, Ar-H), 8.23 (d, *J* = 8.4 Hz, 2H, Ar-H), 8.40 (d, *J* = 8.4 Hz, 2H, Ar-H), 8.67 (d, *J* = 4.8 Hz, 1H, pyrimidine H5) and 8.96 ppm (s, 1H, pyridazine H5); ^13^C-NMR (DMSO-*d_6_*): δ = 93.9, 107.9, 123.5, 126.1, 126.2, 128.7, 128.8, 129.0, 129.3, 130.1, 130.9, 132.1, 136.1, 138.0, 139.9, 140.2, 141.5, 147.9, 149.9, 150.6, 155.0 and 158.3 ppm (Ar-C and CO); MS (EI): *m/z* (%) 487 ([M + 1]^+^, 35.02), 486 (M^+^, 100); HRMS (EI): *m/z* calcd. for C_28_H_18_N_6_O_3_ (M^+^) 486.1434, found 486.1432. Anal. calcd. for C_28_H_18_N_6_O_3_ (486.49): C, 69.13; H, 3.73; N, 17.27. Found C, 69.27; H, 3.65; N, 17.39.

*6-(Benzo[4,5]imidazo[1,2-a]pyrimidine-4-yl)-4-(4-nitrophenyl)-2-phenyl-(2H)-pyridazin-3-one* (**7**). Yellow crystals, yield: (0.6 g, 73%), m.p.: above 300 °C; IR (KBr): *v*/cm^−1^ 1669 (CO); ^1^H-NMR (DMSO-*d_6_*): δ = 7.45 (t, *J* = 7.8 Hz, 1H, Ar-H), 7.54 (t, *J* = 7.8 Hz, 1H, Ar-H), 7.57–762 (m, 3H, Ar-H), 7.74 (d, *J* = 5.4 Hz, 1H, pyrimidine H6), 7.80 (d, *J* = 8.4 Hz, 2H, Ar-H), 7.88 (d, *J* = 7.8 Hz, 1H, Ar-H), 8.24 (d, *J* = 9.0 Hz, 2H, Ar-H), 8.28 (d, *J* = 7.8 Hz, 1H, Ar-H), 8.34 (d, *J* = 9.0 Hz, 2H, Ar-H), 8.69 (s, 1H, pyridazine H5) and 9.48 ppm (d, *J* = 5.4 Hz, 1H, pyrimidine H5); ^13^C-NMR (DMSO-*d_6_*): δ = 102.9, 112.9, 119.5, 119.9, 122.5, 123.7, 126.4, 126.7, 127.8, 129.0, 129.2, 130.7, 136.6, 138.3, 140.7, 142.4, 142.7, 145.2, 148.7, 150.2, 157.0 and 159.1 ppm (Ar-C and CO); MS (EI): *m/z* (%) 412 ([M + 1]^+^, 26.11), 411 (M^+^, 100); HRMS (EI): *m/z* calcd. for C_21_H_13_N_7_O_3_ (M^+^) 411.1074, found 411.1074.

#### 3.2.3. General Procedure for the Preparation of Compounds **16**–**20**

Independent mixtures of **8a** (1.03 g, 2.5 mmol), cyanoacetylindoles **11**,**12**, cyanoacetamides **13**–**15** (2.5 mmol), and ammonium acetate (2 g) in acetic acid (20 mL) were stirred at reflux for 1–2 h. (progress of the reactions monitored by using TLC with 1:1 ethyl acetate/petroleum ether as eluent). The mixtures were cooled to room temperature. The formed solids were collected by filtration and crystallized from the indicated solvents to give **16**–**20** as pure products.

*[2-Amino-6-(4-bromophenyl)-5-(4-chloro-3-nitrophenylazo)pyridin-3-yl](1-methyl-1H-indol-3-yl)-methanone* (**16**). Recrystallized from EtOH/DMF mixture (1:1) as pale brown crystals, yield: (1.10 g, 75%), m.p.: 273–274 °C; IR (KBr): *v*/cm^−1^ 3329, 3279 (NH_2_), 1635( (CO); ^1^H-NMR (DMSO-*d_6_*): δ = 3.89 (s, 3H, CH_3_), 7.32 (t, *J* = 8.0 Hz, 1H, Ar-H), 7.37 (t, *J* = 8.0 Hz, 1H, Ar-H), 7.62 (d, *J* = 8.0 Hz, 1H, Ar-H), 7.74 (d, *J* = 8.4 Hz, 2H, Ar-H), 7.82 (d, *J* = 8.4 Hz, 2H, Ar-H), 7.87 (s, 2H, NH_2_), 7.88–7.93 (m, 2H, Ar-H), 8.13 (s, 1H, indole H2), 8.24 (d, *J* = 8.0 Hz, 1H, Ar-H), 8.30 (s, 1H, Ar-H) and 8.39 ppm (s, 1H, pyridine H4); ^13^C-NMR (DMSO-*d_6_* at 80 °C): δ = 33.7 (CH_3_), 111.2 (pyridine C3), 115.4, 117.3, 119.5, 122.1, 122.8, 123.6, 123.9, 126.2, 127.0, 127.2, 127.5, 131.0, 133.2, 133.4, 137.1, 138.2, 139.3, 145.0, 148.8, 152.0, 159.9, 160.2 and 189.0 ppm (Ar-C and CO); MS (EI): *m/z* (%) 590 ([M + 2]^+^, 37.92), 589 ([M + 1]^+^, 36.11), 588 (M^+^, 26.44); HRMS (EI): *m/z* Calcd. for C_27_H_18_^79^Br^35^ClN_6_O_3_ (M^+^) 588.0306, found 588.0306. Anal. calcd. for C_27_H_18_BrClN_6_O_3_ (589.84): C, 54.98; H, 3.08; N, 14.25. Found C, 54.83; H, 3.17; N, 14.11.

*[2-Amino-6-(4-bromophenyl)-5-(4-chloro-3-nitrophenylazo)pyridin-3-yl](2-methyl-1H-indol-3-yl)- methanone* (**17**). Recrystallized from EtOH/DMF mixture (1:1) as pale brown crystals, yield: (1.16 g, 79%), m.p.: 292–293 °C; IR (KBr): *v*/cm^−1^ 3345, 3267, 3168 (NH_2_ and NH), 1631( (CO); ^1^H-NMR (DMSO-*d_6_*): δ = 2.48 (s, 3H, CH_3_), 7.06 (t, *J* = 8.0 Hz, 1H, Ar-H), 7.15 (t, *J* = 8.0 Hz, 1H, Ar-H), 7.42 (d, *J* = 8.0 Hz, 1H, Ar-H), 7.53 (d, *J* = 8.0 Hz, 1H, Ar-H), 7.73 (d, *J* = 8.4 Hz, 2H, Ar-H), 7.80–7.88 (m, 4H, Ar-H),7.94 (s, 2H, NH_2_), 8.19 (d, *J* = 2.4 Hz, 1H, Ar-H), 8.26 (s, 1H, pyridine H4) and 12.07 ppm (s, 1H, NH); ^13^C-NMR (DMSO-*d_6_*): δ = 14.4 (CH_3_), 111.5 (pyridine C3), 112.7, 117.1, 119.4, 119.9, 121.3, 122.1, 123.2, 125.7, 126.3, 127.1, 127.8, 130.6, 132.7, 133.2, 135.0, 136.1, 136.4, 144.4, 148.0, 151.1, 159.5, 160.0 and 190.2 ppm (Ar-C and CO); MS (EI): *m/z* (%) 590 ([M + 2]^+^, 28.79), 589 ([M + 1]^+^, 26.98), 588 (M^+^, 21.03); HRMS (EI): *m/z* Calcd. for C_27_H_18_^79^BrClN_6_O_3_ (M^+^) 588.0306, found 588.0307.

*2-Amino-6-(4-bromophenyl)-N-(4-chloro-3-nitrophenyl)-5-(4-chloro-3-nitrophenylazo)nicotin-amide* (**18**). Recrystallized from dioxane as orange crystals, yield: (1.27 g, 81%), m.p.: 299–300 °C; IR (KBr): *v*/cm^−1^ 3371, 3266, 3170 (NH_2_ and NH), 1658 (CO); ^1^H-NMR (DMSO-*d_6_*): δ = 7.69 (d, *J* = 8.4 Hz, 2H, Ar-H), 7.74–7.76 (m, 3H, Ar-H), 7.91–7.99 (m, 3H, Ar-H), 8.04 (s, 2H, NH_2_), 8.28 (d, *J* = 2.4 Hz, 1H, Ar-H), 8.50 (d, *J* = 2.4 Hz, 1H, Ar-H), 8.57 (s, 1H, pyridine H4) and 11.00 ppm (s, 1H, NH); ^13^C-NMR (DMSO-*d_6_*): δ = 110.2 (pyridine C3), 117.0, 119.2, 119.3, 123.3, 125.4, 125.6, 126.0, 126.7, 130.5, 131.9, 133.0, 133.1, 135.9, 136.2, 138.7, 147.0, 148.0, 151.1, 159.9, 160.4 and 166.1 ppm (Ar-C and CO); MS (EI): *m/z* (%) 631 ([M + 2]^+^, 66.14), 630 ([M + 1]^+^, 100), 629 (M^+^, 50.07); HRMS (EI): *m/z* Calcd. for C_24_H_14_^79^BrCl_2_N_7_O_5_ (M^+^) 628.9611, found 628.9611. Crystal Data, C_52_H_36_Br_2_Cl_4_N_14_O_12_, M = 1350.57, triclinic, a = 8.0796(5) Å, b = 11.0560(7) Å, c = 16.1457(11) Å, V = 1336.71(15) Å^3^, α = 72.539(5)°, β = 76.338(5)°, γ = 86.790(6)°, space group: P-1, Z = 1, D_calc_ = 1.678 g·cm^−3^, No. of reflection measured 6079, 2 θ _max_ = 27.44°, R1 = 0.0386 [42].

*N-(5-Acetyl-2-phenyl-2H-1,2,3-triazol-4-yl)-2-amino-6-(4-bromophenyl)-5-(4-chloro-3-nitrophenyl)-nicotinamide* (**19**). Recrystallized from dioxane as deep orange crystals, yield: (1.19 g. 72%), m.p.: above 300 °C; IR (KBr): *v*/cm^−1^ 3382, 3351, 3166 (NH_2_ and NH), 1689, 1659 ( (2CO); ^1^H-NMR (DMSO-*d_6_*): δ = 2.66 (s, 3H, CH_3_), 7.54 (t, *J* = 7.6 Hz, 1H, Ar-H), 7.65 (t, *J* = 7.6 Hz, 2H, Ar-H), 7.72 (d, *J* = 8.4 Hz, 2H, Ar-H), 7.79–7.82 (m, 2H, Ar-H), 7.99 (s, 2H, NH_2_), 8.07–8.10 (m, 4H, Ar-H), 9.30 (s, 1H, Ar-H), 8.74 (s, 1H, pyridine H4) and 11.21 ppm (s, 1H, NH); MS (EI): *m/z* (%) 661 ([M + 2]^+^, 77.95), 660 ([M + 1]^+^, 100), 659 (M^+^, 56.01); HRMS (EI): *m/z* Calcd. for C_28_H_19_^79^BrClN_9_O_4_ (M^+^) 659.0426, found 659.0428.

*6-(4-Bromobenzoyl)-2-(4-chloro-3-nitrophenyl)-3-imino-2,3-dihydropyridazine-4-carboxylic acid (3-cyano-4-phenylthiophen-2-yl)amide* (**20a**). Recrystallized from EtOH/DMF mixture (1:3) as pale brown crystals, yield: (1.27 g, 77%), m.p.: 270–271 °C; IR (KBr): *v*/cm^−1^ 3426, 3346 (2 NH), 2216 (CN), 1678, 1659 (CO); ^1^H-NMR (DMSO-*d_6_*): δ = 7.16 (s, 1H, thiophene H5), 7.37 (t, *J* = 8.0 Hz, 1H, Ar-H), 7.47 (t, *J* = 8.0 Hz, 2H, Ar-H), 7.63 (d, *J* = 8.0 Hz, 2H, Ar-H), 7.75 (d, *J* = 8.8 Hz, 2H, Ar-H), 7.95 (d, *J* = 8.8 Hz, 2H, Ar-H), 8.16–8.23 (m, 2H, Ar-H), 8.73 (d, *J* = 2.0 Hz, 1H, Ar-H), 8.79 (s, 1H, pyridazine H5), 9.85 (s, 1H, imine NH) and 13.32 ppm (s, 1H, amide NH); ^13^C-NMR (DMSO-*d_6_*): δ = 94.4 (thiophene C-3), 114.9, 117.8, 125.3, 127.0, 127.7, 128.40, 128.42, 128.8, 130.9, 131.2, 131.7, 132.6, 132.8, 133.4, 134.51, 134.54, 136.8, 138.8, 146.3, 148.2, 155.0, 161.0, 163.4 and 187.4 ppm (Ar-C, CN and CO); MS (EI): *m/z* (%) 660 ([M + 2]^+^, 7.95), 659 ([M + 1]^+^, 2.73), 658 (M^+^, 6.55); HRMS (EI): *m/z* Calcd. for C_29_H_16_^79^BrClN6O_4_S (M^+^) 657.9820, found 657.9820.

6*-(4-Bromobenzoyl)-2-(4-chloro-3-nitrophenyl)-3-imino-2,3-dihydropyridazine-4-carboxylic acid [4-(4-chlorophenyl)-3-cyanothiophen-2-yl]amide* (**20b**). Recrystallized from EtOH/DMF mixture (1:3) as yellowish brown crystals, yield: (1.26 g, 73%), m.p.: 288–289 °C; IR (KBr): *v*/cm^−1^ 3401, 3349 (2 NH), 2214 (CN), 1673, 1654 (CO); ^1^H-NMR (DMSO-*d_6_*): δ = 7.16 (s, 1H, thiophene H5), 7.53 (d, *J* = 8.8 Hz, 2H, Ar-H), 7.65 (d, *J* = 8.4 Hz, 2H, Ar-H), 7.76 (d, *J* = 8.8 Hz, 2H, Ar-H), 7.95 (d, *J* = 8.4 Hz, 2H, Ar-H), 8.17–8.19 (m, 2H, Ar-H), 8.71 (d, *J* = 2.0 Hz, 1H, Ar-H), 8.78 (s, 1H, pyridazine H5), 9.84 (s, 1H, imine NH) and 13.25 ppm (s, 1H, amide NH); ^13^C-NMR (DMSO-*d_6_*): δ = 94.1 (thiophene C-3), 115.5, 117.6, 125.2, 128.4, 128.7, 128.8, 131.0, 131.2, 131.7, 132.4, 132.6, 132.8, 133.36, 133.39, 134.5, 135.4, 138.8, 146.3, 148.1, 155.0, 161.1, 163.7 and 187.5 ppm (Ar-C, CN and CO); MS (EI): *m/z* (%) 694 ([M + 2]^+^, 11.84), 693 ([M + 1]^+^, 5.12), 692 (M^+^, 8.92); HRMS (EI): *m/z* Calcd. for C_29_H_15_^79^BrCl_2_N6O_4_S (M^+^) 691.9430, found 691.9432.

#### 3.2.4. General Procedure for the Preparation of Naphthyridine Derivatives **24a**,**b**

Independent solutions of cyanoacetic acid (0.425 g, 5 mmol) in Ac_2_O (10 mL) was heated at 100 °C for 5 min. then compounds **10a**,**b** (5 mmol) were added and the reaction mixtures were heated for further 30 min. at 100 °C. Then the reaction mixtures were allowed to cool to room temperature and the formed crystalline solids were separated by filtration, washed with ethanol and recrystallized from the proper solvent.

*7-(4-Bromophenyl)-6-(4-chloro-3-nitrophenylazo)-2-oxo-4-phenyl-1,2-dihydro[1,8]naphthyridine-3-carbonitrile* (**24a**).Recrystallized from EtOH/dioxane mixture (1:1) as pale brown crystals, yield: (2.54 g, 80%), m.p.: above 300 °C; IR (KBr): *v*/cm^−1^ 3397 (NH), 2220 (CN), 1673 (CO); ^1^H-NMR (DMSO-*d_6_*): δ = 7.51–7.53 (m, 2H, Ar-H), 7.62–7.63 (m, 3H, Ar-H), 7.71 (d, *J* = 8.4 Hz, 2H, Ar-H), 7.77 (d, *J* = 8.4 Hz, 2H, Ar-H), 7.80–7.89 (m, 3H, 2 Ar-H and pyridine H4), 8.18 (d, *J* = 2.4 Hz, 1H, Ar-H) and 13.33 ppm (s, 1H, NH); ^13^C-NMR (DMSO-*d_6_*): δ = 107.3, 113.5, 117.7, 120.0, 122.5, 123.6, 126.6, 127.0, 129.3, 129.5, 130.1, 131.1, 133.2, 133.6, 134.5, 137.5, 138.7, 145.6, 148.5, 151.4, 157.9, 158.0 and 160.7 ppm (Ar-C, CN and CO); MS (EI): *m/z* (%) 586 ([M + 2]^+^, 61.03), 585 ([M + 1]^+^, 84.11), 584 (M^+^, 47.05); HRMS (EI): *m/z* calcd. for C_27_H_14_^79^Br^35^ClN_6_O_3_ (M^+^) 583.9993, found 583.9991. Anal. calcd. for C_27_H_14_BrClN_6_O_3_ (585.81): C, 55.36; H, 2.41; N, 14.35. Found C, 55.24; H, 2.49; N, 14.27.

*[7-(4-Chlorophenyl)-6-(4-chloro-3-nitrophenylazo)-2-oxo-4-phenyl-1,2-dihydro[1,8]naphthyridine-3-carbonitrile* (**24b**). Recrystallized from EtOH/dioxane mixture (1:1) as pale brown crystals, yield: (2.21 g, 82%), m.p.: above 300 °C; IR (KBr): *v*/cm^−1^ 3372 (NH), 2228 (CN), 1672 (CO); ^1^H-NMR (DMSO-*d_6_*): δ = 7.56–7.61 (m, 4H, Ar-H), 7.65–7.68 (m, 3H, Ar-H), 7.81–7.85 (m, 3H, Ar-H), 7.87–7.91 (m, 2H, 1 Ar-H and pyridine H4), 8.23 (d, *J* = 2.4 Hz, 1H, Ar-H) and 13.45 ppm (s, 1H, NH); ^13^C-NMR (DMSO-*d_6_*): δ = 108.2, 113.6, 114.8, 120.2, 123.9, 126.7, 127.7, 128.1, 128.8, 129.1, 130.6, 132.3, 132.9, 133.1, 134.7, 135.4, 140.6, 148.0, 150.3, 151.3, 159.3, 159.45 and 159.52 ppm (Ar-C, CN and CO); MS (EI): *m/z* (%) 541 ([M + 1]^+^, 88.31), 540 (M^+^, 100); HRMS (EI): *m/z* calcd. for C_27_H_14_^35^Cl_2_N_6_O_3_ (M^+^) 540.0498, found 540.0495.

## 4. Conclusions

The results of the study described above have led to the development of a simple approach for the synthesis of a novel class of pyridazin-3-one and 2-aminopyridine derivatives. Furthermore, the observations made during this work showed that these compounds are versatile precursors for the synthesis of some very important fused azines like azolo[1,5-*a*]pyrimidines and 1,8-naphthyridines, for which we expect a wide range of biological activity.

## Supplementary Materials

Supplementary materials can be accessed at: http://www.mdpi.com/1420-3049/19/2/2637/s1.

## References

[1] Zhmurenko L.A., Molodavkin G.M., Voronina T.A., Lezina V.P. (2012). Synthesis and antidepressant and anxiolytic activity of derivatives of pyrazolo[4,3-*c*]pyridine and 4-phenylhydrazinonicotinic acids. Pharm. Chem. J..

[2] Smyth L.A., Matthews T.P., Horton P.N., Hursthouse M.B., Collins I. (2010). Synthesis and reactivity of 3-amino-1H-pyrazolo[4,3-*c*]pyridin-4(5*H*)-ones: Development of a novel kinase-focussed library. Tetrahedron.

[3] Suksrichavalit T., Prachayasittikul S., Nantasenamat C., Isarankura-Na-Ayudhya C., Prachayasittikul V. (2009). Copper complexes of pyridine derivatives with superoxide scavenging and antimicrobial activities. Eur. J. Med. Chem..

[4] Gaonkar S.L., Rai K.M.L., Prabhuswamy B. (2007). Synthesis of novel 3-[5-ethyl-2-(2-phenoxy-ethyl)-pyridin]-5-substituted isoxazoline libraries via 1,3-dipolar cycloaddition and evaluation of antimicrobial activities. Med. Chem. Res..

[5] Butnariu R.M., Caprosu M.D., Bejan V., Mangalagiu I.I., Ungureanu M., Poiata A., Tuchilus C., Florescu M. (2007). Pyridazine and phthalazine derivatives with potential antimicrobial activity. J. Heterocycl. Chem..

[6] Kandile N.G., Mohamed M.I., Zaky H., Mohamed H.M. (2009). Novel pyridazine derivatives: Synthesis and antimicrobial activity evaluation. Eur. J. Med. Chem..

[7] Hosni H.M., Abdulla M.M. (2008). Anti-inflammatory and analgesic activities of some newly synthesized pyridinedicarbonitrile and benzopyranopyridine derivatives. Acta Pharm..

[8] Dogruer D.S., Unlu S., Kupeli E., Banoglu E., Sahin M.F. (2007). Synthesis of 2-[5,6-diphenyl-3(2*H*)-pyridazinone-2-yl]acetamide and 3-[5,6-diphenyl-3(2*H*)-pyridazinone-2-yl]propanamide derivatives as analgesic and anti inflamematory agents. Turk. J. Pharm. Sci..

[9] Gökçe M., Colak M.S., Küpeli E., Sahin M.F. (2009). Synthesis and analgesic and anti-inflammatory activity of 6-phenyl/(4-methylphenyl)-3(2*H*)-pyridazinon-2-propionamide derivatives. Arzneim. Forsch..

[10] Ali M.A., Yar M.S., Siddiqui A.A., Sriram D., Yogeeswari P., de Clercq E. (2007). Synthesis and anti-HIV activity of *N'*-nicotinoyl-3-(4'-hydroxy-3'-methylphenyl)-5-[substituted phenyl]-2-pyrazo- lines. Acta Pol. Pharm..

[11] Kumar S., Das S.K., Dey S., Maity P., Guha M., Choubey V., Panda G., Bandyopadhyay U. (2008). Antiplasmodial activity of [(aryl)arylsulfanylmethyl]pyridine. Antimicrob. Agents Chemother..

[12] Lourenço M.C.S., de Souza M.V.N., Pinheiro A.C., de Lima Ferreira M., Goncalves R.S.B., Nogueira T.C.M., Peralta M.A. (2007). Evaluation of anti-tubercular activity of nicotinic and isoniazid analogues. ARKIVOC.

[13] Sharma P.C., Jain S. (2008). Synthesis and *in vitro* antibacterial activity of some novel *N*-nicotinoyl-1-ethyl-6-fluoro-1,4-dihydro-7-piperazin-1-yl-4-oxoquinoline-3-carboxylates. Acta Pol. Pharm..

[14] Shafiee A., Rastkari N., Sharifzadeh M. (2004). Anticonvulsant activities of new 1,4-dihydropyridine derivatives containing 4-nitroimidazolyl substituents. Daru.

[15] Rubat C., Coudert P., Refouvelet B., Tronche P., Bastide P., Bastide J. (1990). Anticonvulsant activity of 3-oxo-5-substituted benzylidene-6-methyl-(4*H*)-2-pyridazinylacetamides and 2-pyridazinylacetyl-hydrazides. Chem. Pharm. Bull..

[16] Chintakunta V.K., Akella V., Vedula M.S., Mamnoor P.K., Mishra P., Casturi S.R., Vangoori A., Rajagopalan R. (2002). 3-*O*-Substituted benzyl pyridazinone derivatives as COX inhibitors. Eur. J. Med. Chem..

[17] Rathish I.G., Javed K., Bano S., Ahmad S., Alam M.S., Pillai K.K. (2009). Synthesis and blood glucose lowering effect of novel pyridazinone substituted benzenesulfonylurea derivatives. Eur. J. Med. Chem..

[18] Barbaro R., Betti L., Botta M., Corelli F., Giannaccini G., Maccari L., Manetti F., Strappaghetti G., Corsano S. (2001). Synthesis, biological evaluation, and pharmacophore generation of new pyridazinone derivatives with affinity toward α_1_- and α_2_-adrenoceptors. J. Med. Chem..

[19] Joule J.A., Mills K. (2010). Heterocyclic Chemistry.

[20] Atanasova M., Ilieva S., Galabov B. (2007). QSAR analysis of 1,4-dihydro-4-oxo-1-(2-thiazolyl)-1,8-naphthyridines with anticancer activity. Eur. J. Med. Chem..

[21] Miguel F.B., Monica C.M.L.G., Elena P.M., Berta L., de Beatriz P.T., Ana R., Nuria A., Francisco L., Dolores M.M., Olivier L. (2005). Pyrazolo[3,4-*c*]pyridazines as novel and selective inhibitors of cyclin-dependent kinases. J. Med. Chem..

[22] Malinka W., Redzicka A., Lozach O. (2004). New derivatives of pyrrolo[3,4-*d*]pyridazinone and their anticancer effects. Farmaco.

[23] Thapa P., Karki R., Thapa U., Jahng Y., Jung M.-J., Nam J.M., Na Y., Kwon Y., Lee E.-S. (2010). 2-Thienyl-4-furyl-6-aryl pyridine derivatives: Synthesis, topoisomerase I and II inhibitory activity, cytotoxicity, and structure–activity relationship study. Bioorg. Med. Chem..

[24] Thapa P., Karki R., Choi H., Choi J.H., Yun M., Jeong B.-S., Jung M.-J., Nam J.M., Na Y., Cho W.-J. (2010). Synthesis of 2-(thienyl-2-yl or -3-yl)-4-furyl-6-aryl pyridine derivatives and evaluation of their topoisomerase I and II inhibitory activity, cytotoxicity, and structure activity relationship. Bioorg. Med. Chem..

[25] Sotelo E., Fraiz N., Yanez M., Terrades V., Laguna R., Cano E., Ravina E. (2002). Pyridazines. Part XXIX: Synthesis and platelet aggregation inhibition activity of 5-substituted-6-phenyl-3(2*H*)-pyridazinones novel aspects of their biological action. Bioorg. Med. Chem..

[26] Griebel G., Perrault G., Sanger D.J. (1999). Differences in anxiolytic-like profile of two novel nonbenzodiazepine BZ (omega) receptor agonists on defensive behaviors of mice. Pharmacol. Biochem. Behav..

[27] Wermuth C.G., Schlewer G., Bourguignon J.J., Maghioros G., Bouchet M.J., Moire C., Kan J.P., Worms P., Biziere K. (1989). 3-Aminopyridazine derivatives with atypical antidepressant, serotonergic, and dopaminergic activities. J. Med. Chem..

[28] Caliskan E.B., Sukuroglu M., Coban T., Banoglu E., Suzen S. (2008). Screening and evaluation of antioxidant activity of some pyridazine derivatives. J. Enz. Inhib. Med. Chem..

[29] Chen K., Kuo S.-C., Hsieh M.-C., Mauger A., Lin C.M., Hamel E., Lee K.-H. (1997). Antitumor agents. 178. Synthesis and biological evaluation of substituted 2-aryl-1,8-naphthyridin-4(1*H*)-ones as antitumor agents that inhibit tubulin polymerization. J. Med. Chem..

[30] Zhang S.-X., Bastow K.F., Tachibana Y., Kuo S.-C., Hamel E., Mauger A., Narayanan V.L., Lee K.-H. (1999). Antitumor agents. 196. Substituted 2-thienyl-1,8-naphthyridin-4-ones: Their synthesis, cytotoxicity, and inhibition of tubulin polymerization. J. Med. Chem..

[31] Wu J., Kang S., Luo L., Shi Q., Ma J., Yin J., Song B., Hu D., Yang S. (2013). Synthesis and antifungal activities of novel nicotinamide derivatives containing 1,3,4-oxadiazole. Chem. Cent. J..

[32] Behbehani H., Ibrahim H.M., Elnagdi M.H. (2013). Non-concerted nucleophilic [4+1] cycloaddition of (dimethylamino)methoxycarbene to arylazonicotinates in the synthesis of pyrazolo[3,4-*c*]pyridines and pyrazolo[4',3':4,5]pyrido[2,3-*d*]pyrimidines. Tetrahedron.

[33] Behbehani H., Ibrahim H.M., Makhseed S., Mahmoud H. (2011). Applications of 2-arylhydrazononitriles in synthesis: Preparation of new indole containing 1,2,3-triazole, pyrazole and pyrazolo[1,5-*a*]pyrimidine derivatives and evaluation of their antimicrobial activities. Eur. J. Med. Chem..

[34] Behbehani H., Ibrahim H.M., Makhseed S., Elnagdi M.H., Mahmoud H. (2012). 2-Aminothiophenes as building blocks in heterocyclic synthesis: Synthesis and antimicrobial evaluation of a new class of pyrido[1,2-*a*]thieno[3,2-*e*]pyrimidine, quinoline and pyridin-2-one derivatives. Eur. J. Med. Chem..

[35] Behbehani H., Ibrahim H.M. (2012). 4-Thiazolidinones in heterocyclic synthesis: Synthesis of novel enaminones, azolopyrimidines and 2-arylimino-5-arylidene-4-thiazolidinones. Molecules.

[36] Behbehani H., Ibrahim H.M. (2013). Organocatalysis in heterocyclic synthesis: DABCO as a mild and efficient catalytic system for the synthesis of a novel class of quinazoline, thiazolo[3,2-*a*]quinazoline and thiazolo[2,3-*b*]quinazoline derivatives. Chem. Cent. J..

[37] Ibrahim H.M., Behbehani H., Elnagdi M.H. (2013). Approaches towards the synthesis of a novel class of 2-amino-5-arylazonicotinate, pyridazinone and pyrido[2,3-*d*]pyrimidine derivatives as potent antimicrobial agents. Chem. Cent. J..

[B38-molecules-19-02637] 38.Crystallographic data for **3d** (ref. CCDC 982246) can be obtained on request from the director, Cambridge Crystallographic Data Center, 12 Union Road, Cambridge CB2 1EW, UK.

[B39-molecules-19-02637] 39.Crystallographic data for **3l** (ref. CCDC 982247) can be obtained on request from the director, Cambridge Crystallographic Data Center, 12 Union Road, Cambridge CB2 1EW, UK.

[40] Behbehani H., Ibrahim H.M. (2013). A strategy for the synthesis of 2-aryl-3-dimethylaminopyrazolo-[3,4-*c*]pyridines that utilizes [4+1] cycloaddition reactions of 5-arylazo-2,3,6-trisubstituted pyridines. Tetrahedron..

[41] Al-Mousawi S.M., Moustafa M.S., Abdelshafy I.A., Elnagdi M.H. (2011). Reassignment of the structures of condensation products of α-keto α'-formylarylhydrazones with ethyl cyanoacetate: A novel route to ethyl 5-arylazo-2-hydroxynicotinates. Tetrahedron Lett..

[B42-molecules-19-02637] 42.Crystallographic data for **18** (ref. CCDC 943164) can be obtained on request from the director, Cambridge Crystallographic Data Center, 12 Union Road, Cambridge CB2 1EW, UK.

[43] Sheldrick G.M. (2008). A short history of *SHELX*. Acta Cryst..

