# Supplementary material for: Synthesis of A New Class of Pyridazin-3-one and 2-Amino-5-arylazopyridine Derivatives and Their Utility in the Synthesis of Fused Azines
Source: Molecules. 2014 Feb 24;19(2):2637–54. doi: 10.3390/molecules19022637 (PMC6272007; doi:10.3390/molecules19022637)

Supplementary Materials

Figure S1. ^{1}H-NMR spectra of compound **16**.

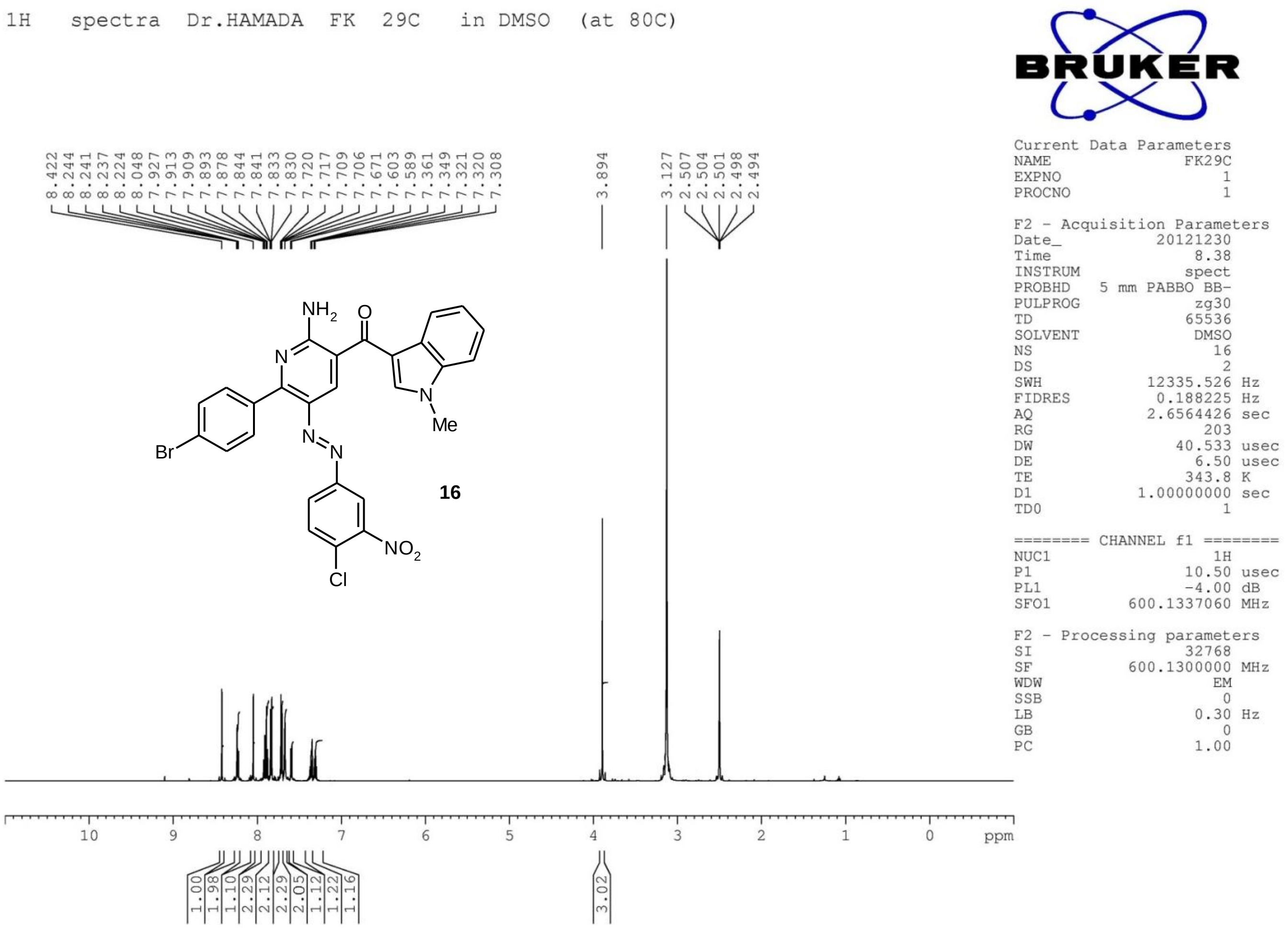

Figure S2. ^{1}H-NMR expansion of compound **16**.

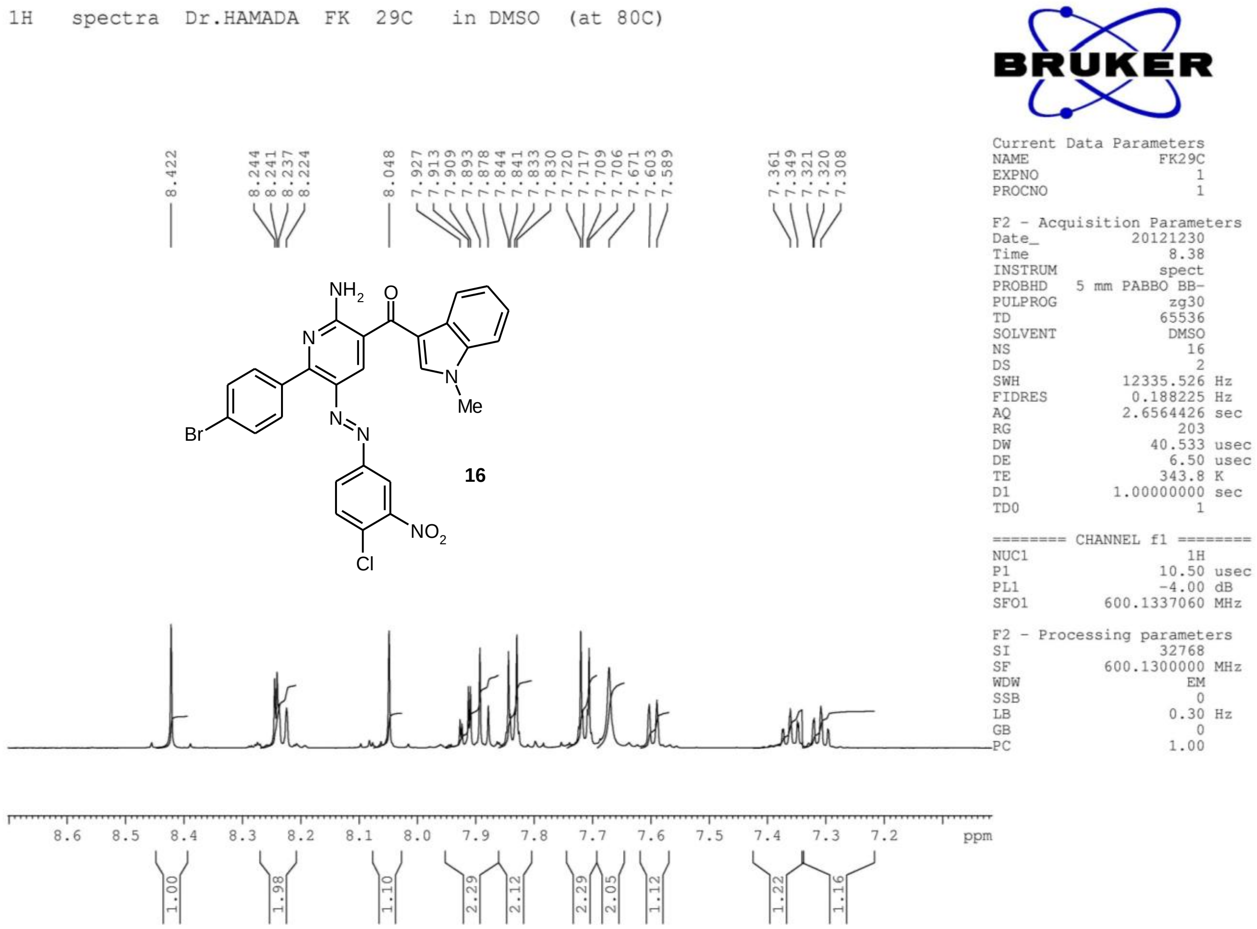

Figure S3. ^{13}C-NMR spectra of compound **16**.

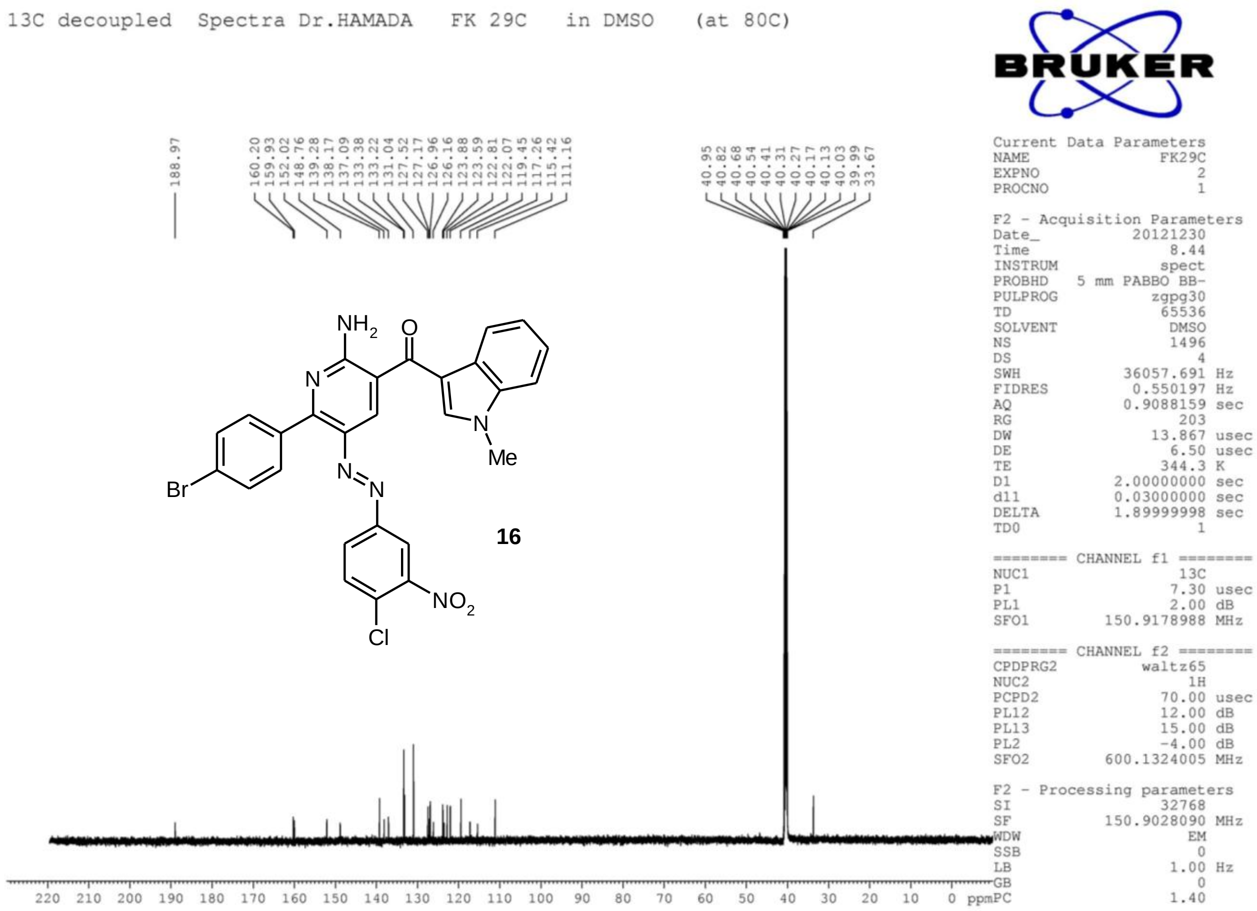

Figure S4. ^{1}H-NMR spectra of compound **17**.

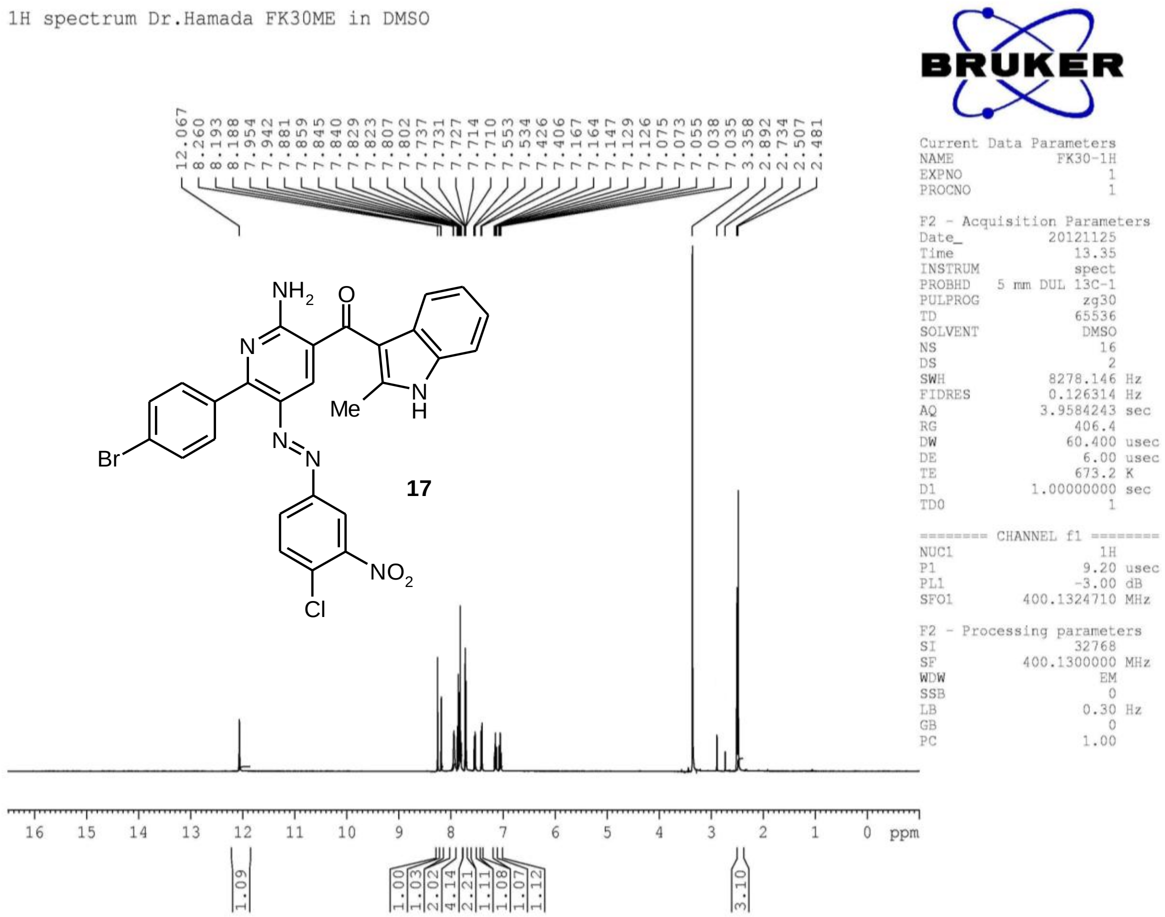

Figure S5. ^{1}H-NMR expansion of compound **17**.

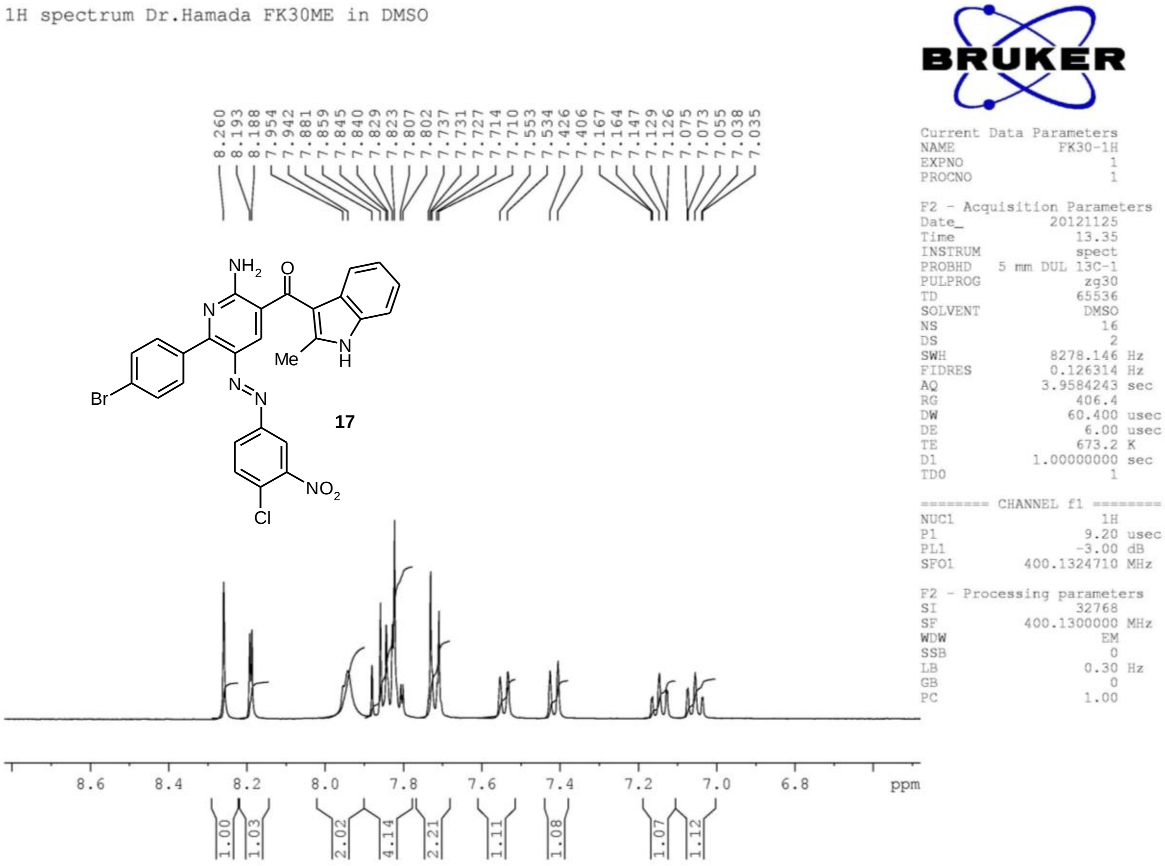

Figure S6. ^{13}C-NMR spectra of compound **17**.

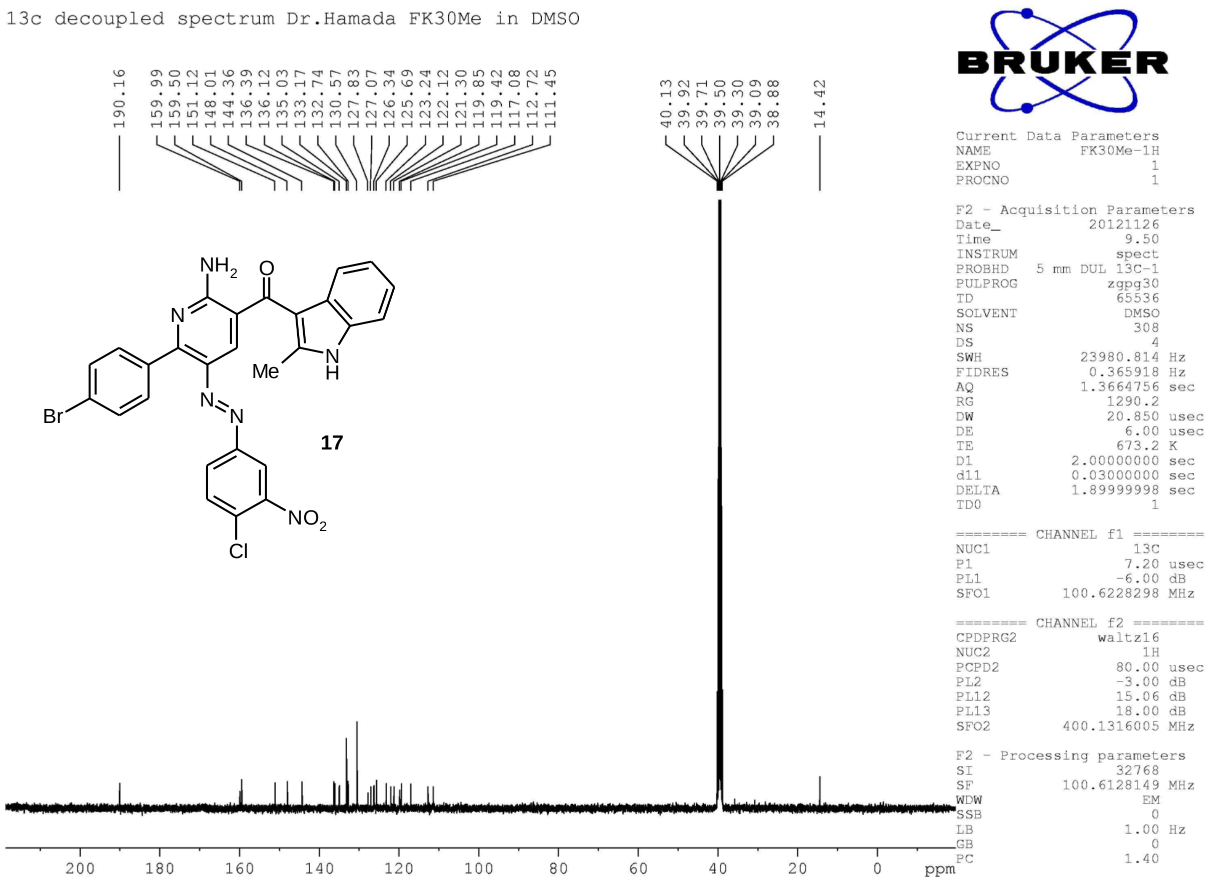

Figure S7. ^{1}H-NMR spectra of compound **18**.

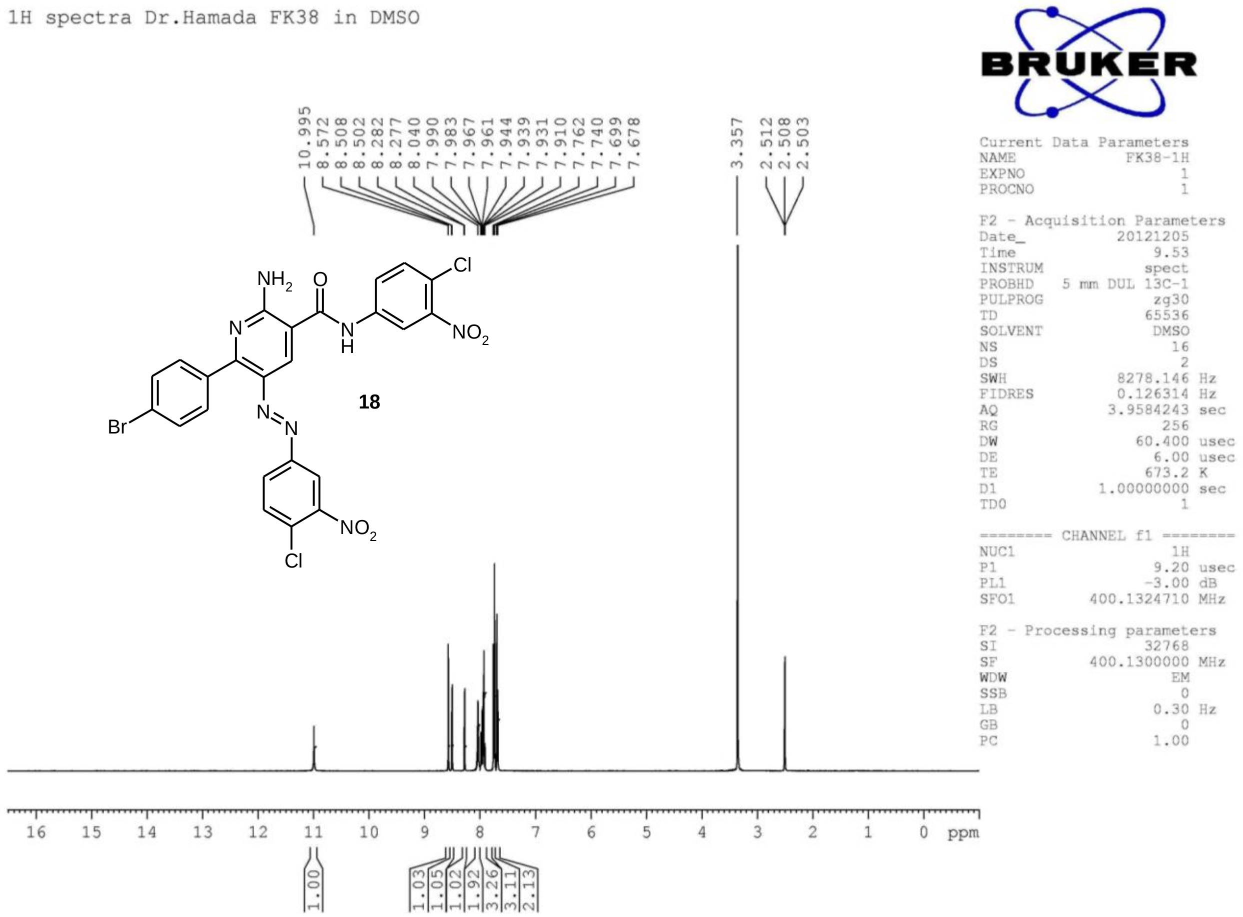

Figure S8. ^{1}H-NMR expansion of compound **18**.

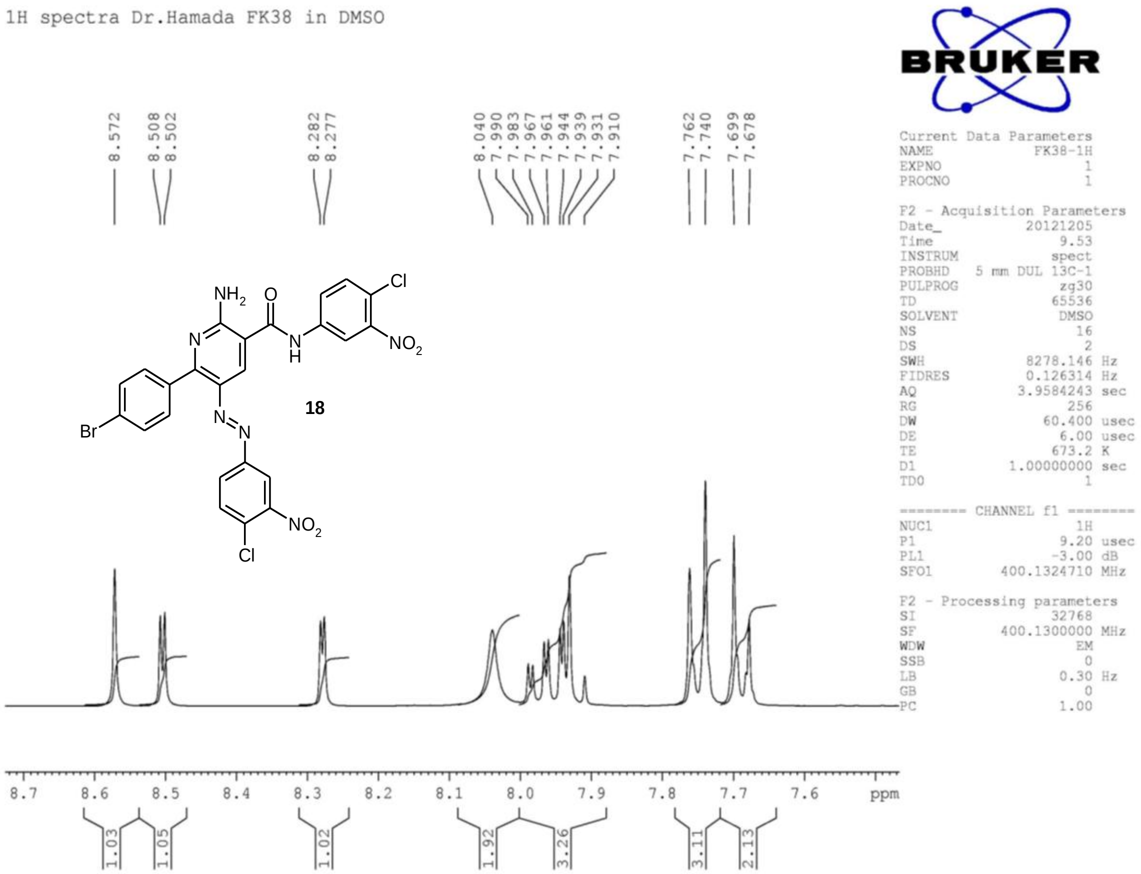

Figure S9. ^{13}C-NMR spectra of compound **18**.

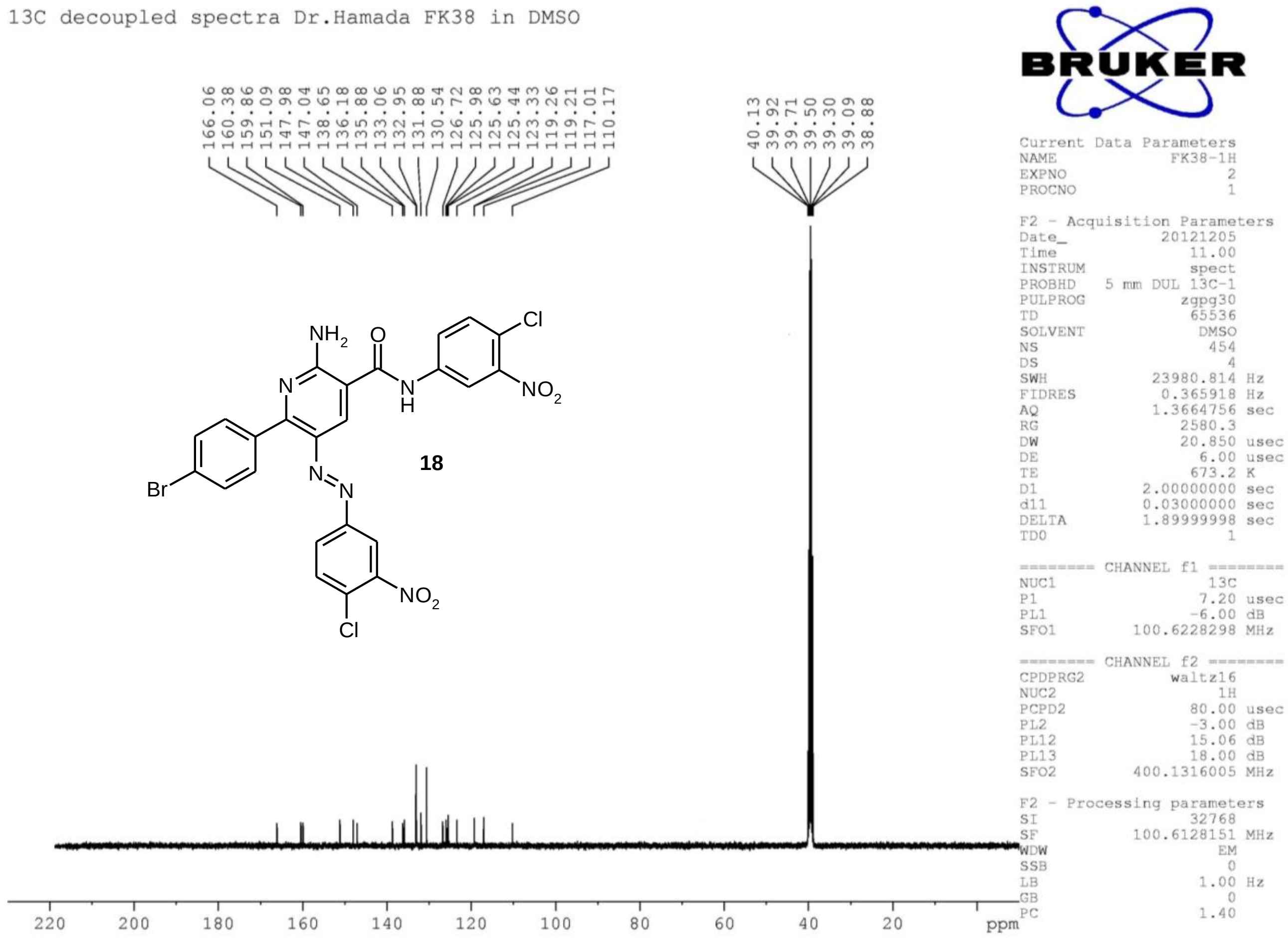

Figure S10. ^{1}H-NMR spectra of compound **20a**.

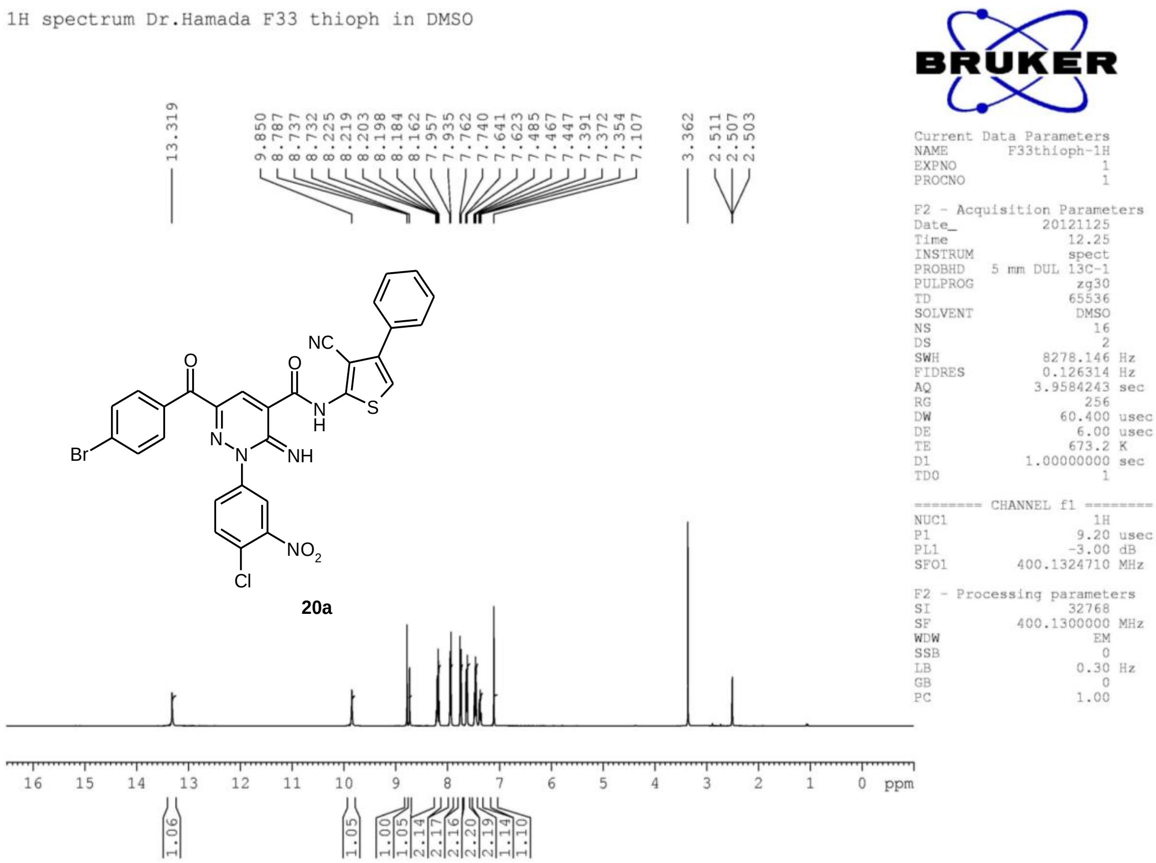

Figure S11. ^{1}H-NMR expansion of compound **20a**.

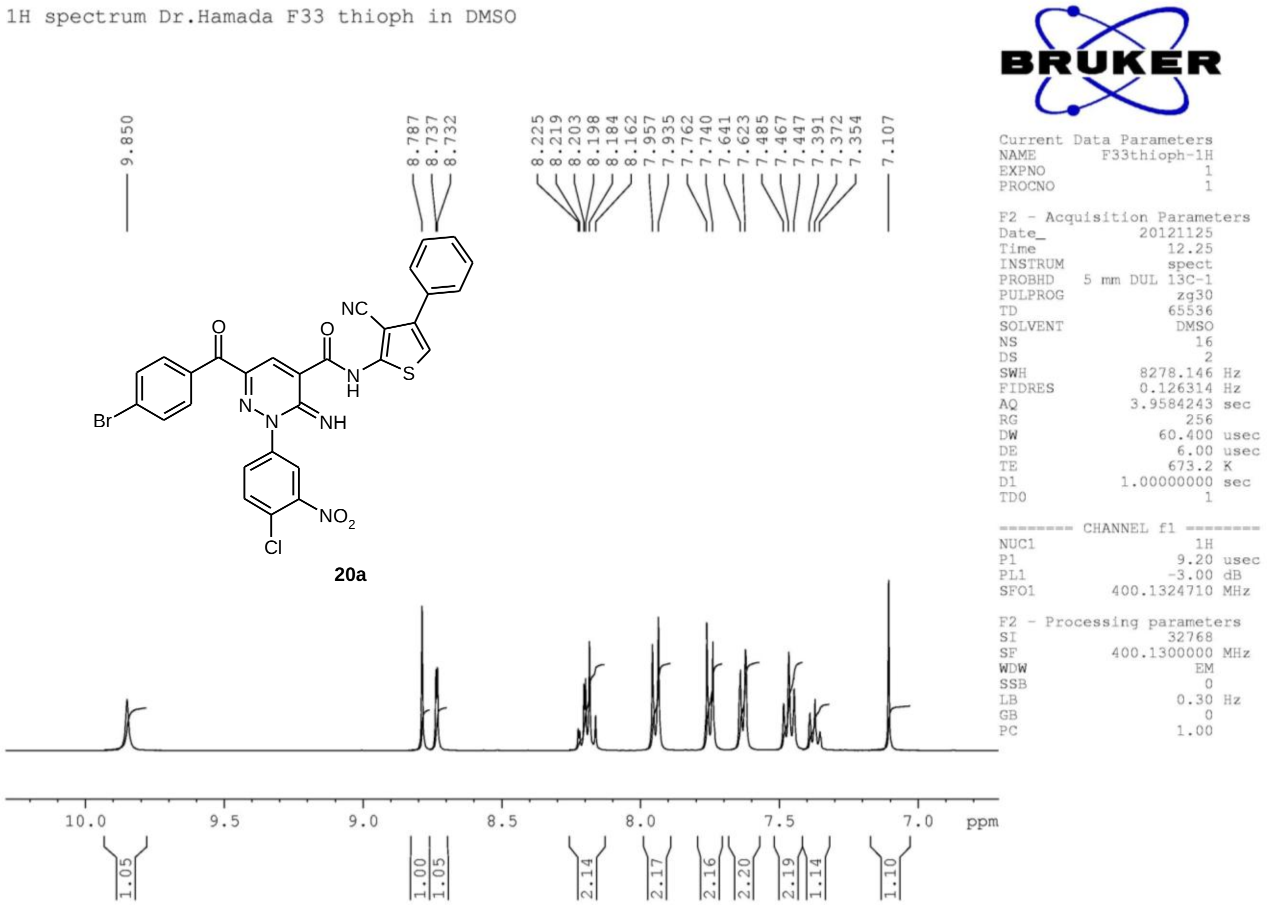

Figure S12. ^{13}C-NMR spectra of compound **20a**.

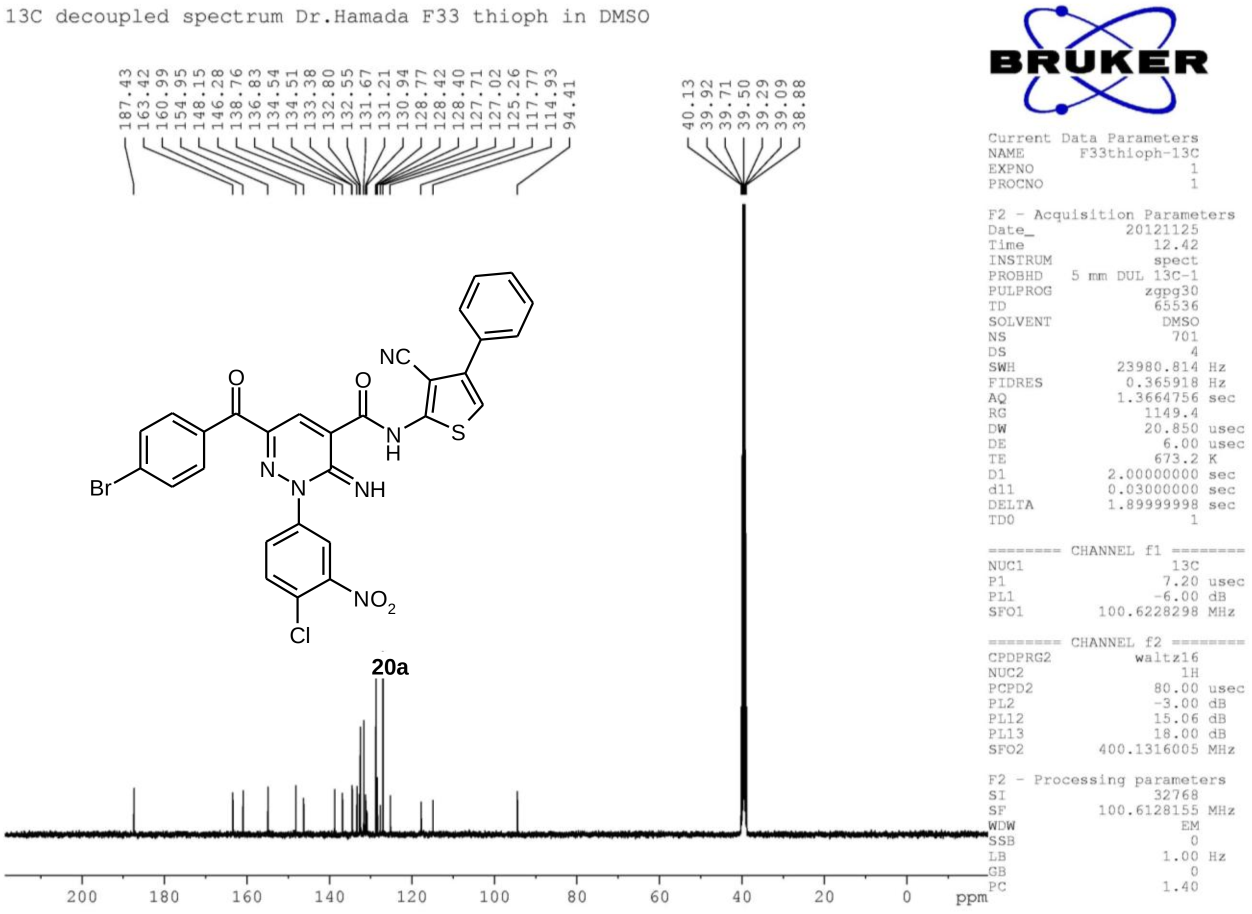

Figure S13. ^{1}H-NMR spectra of compound **20b**.

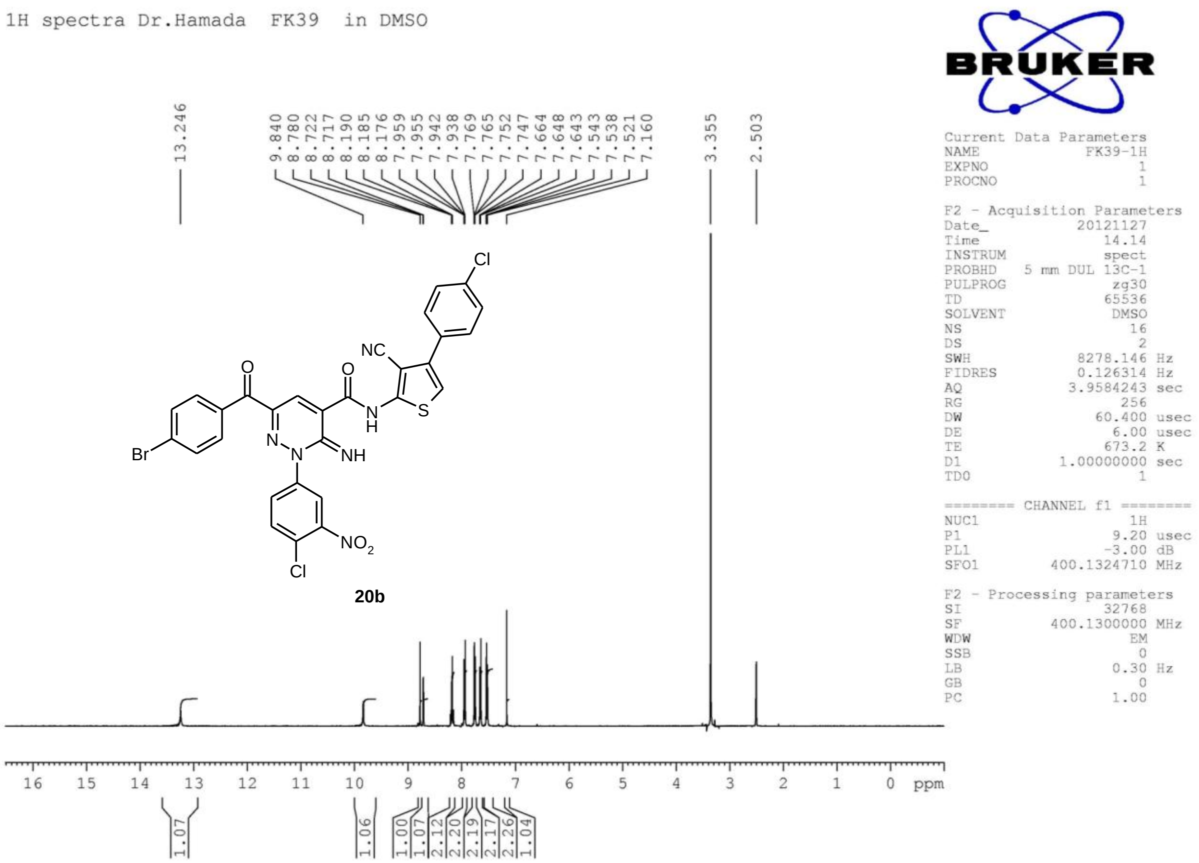

Figure S14. ^{1}H-NMR expansion of compound **20b**.

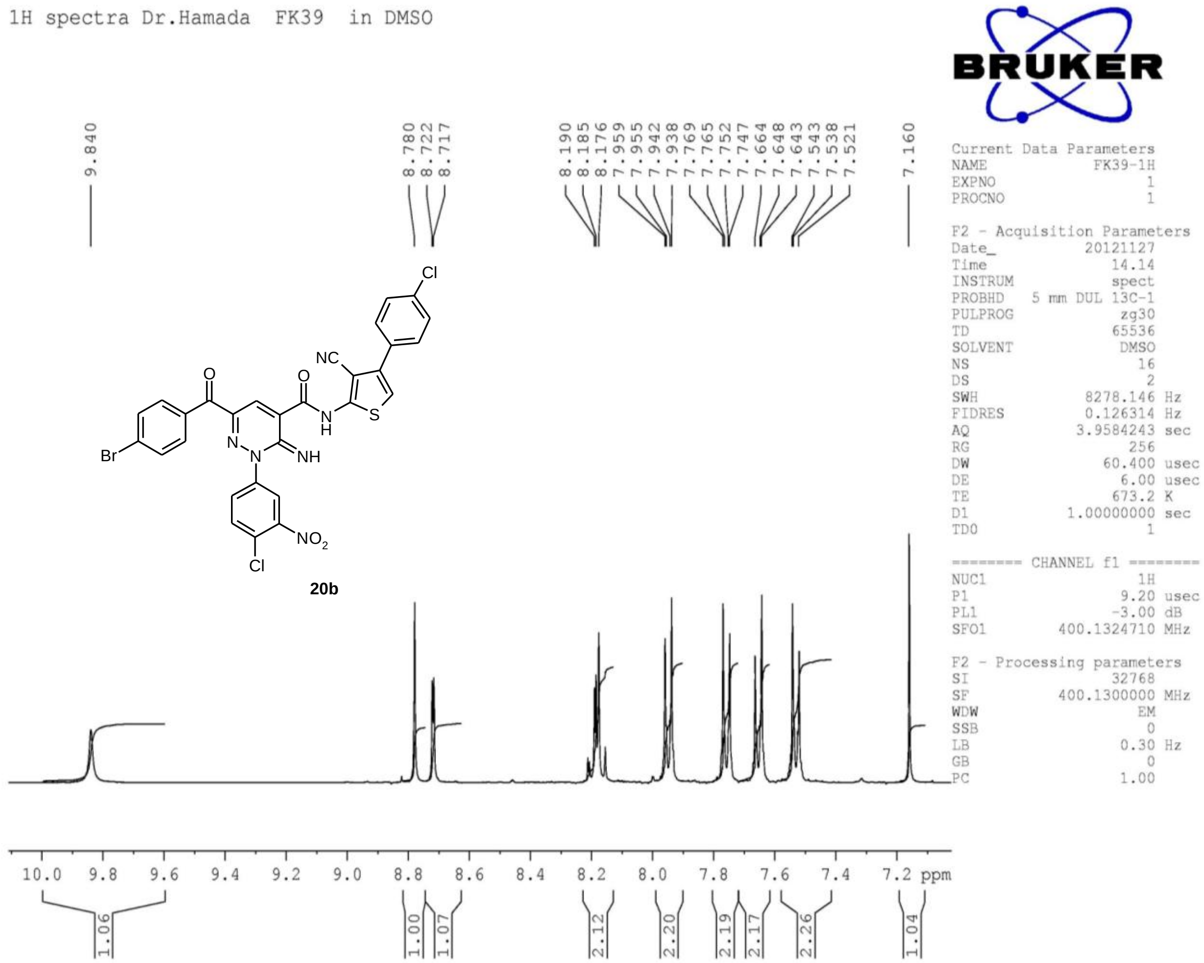

Figure S15. ^{13}C-NMR spectra of compound **20b**.

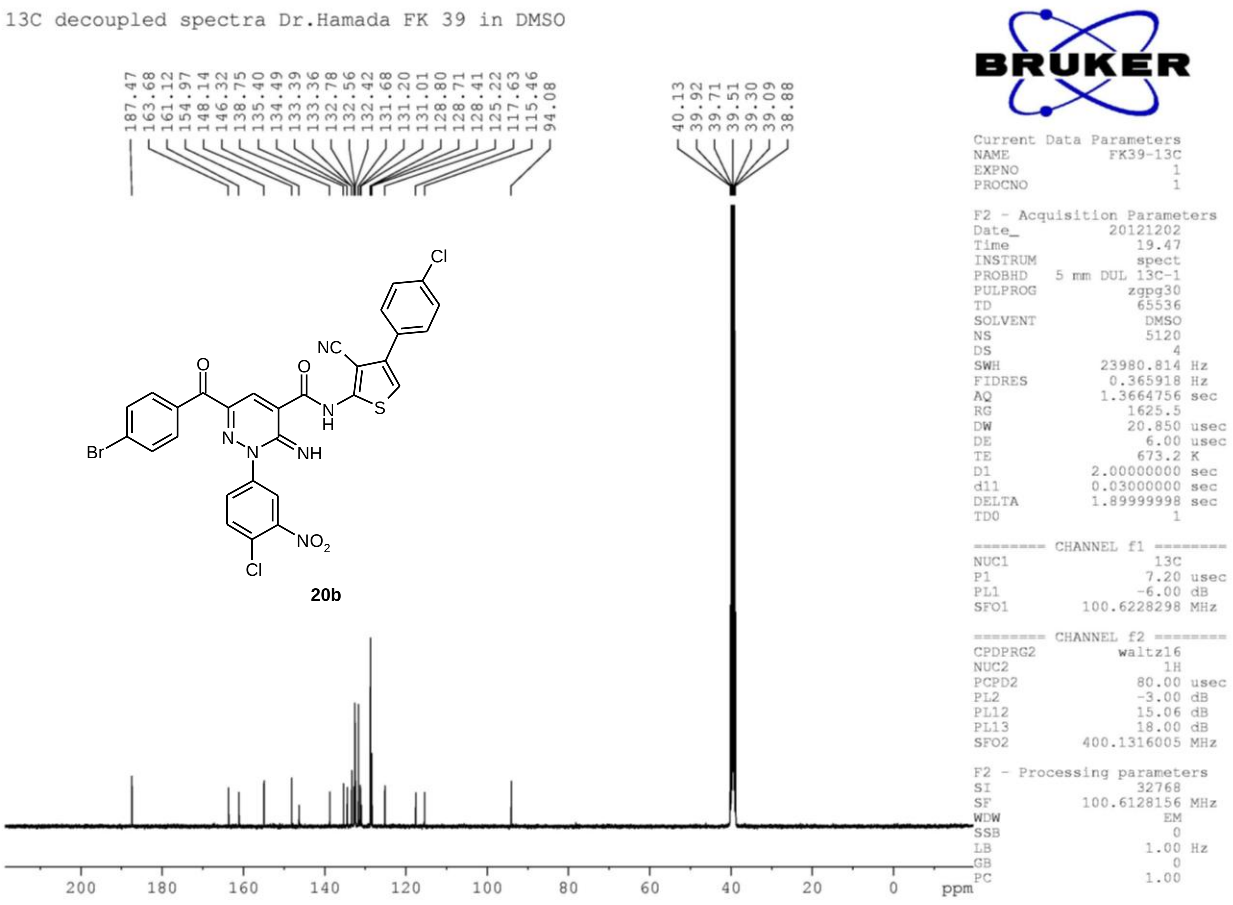

Figure S16. ^{1}H-NMR spectra of compound **24a**.

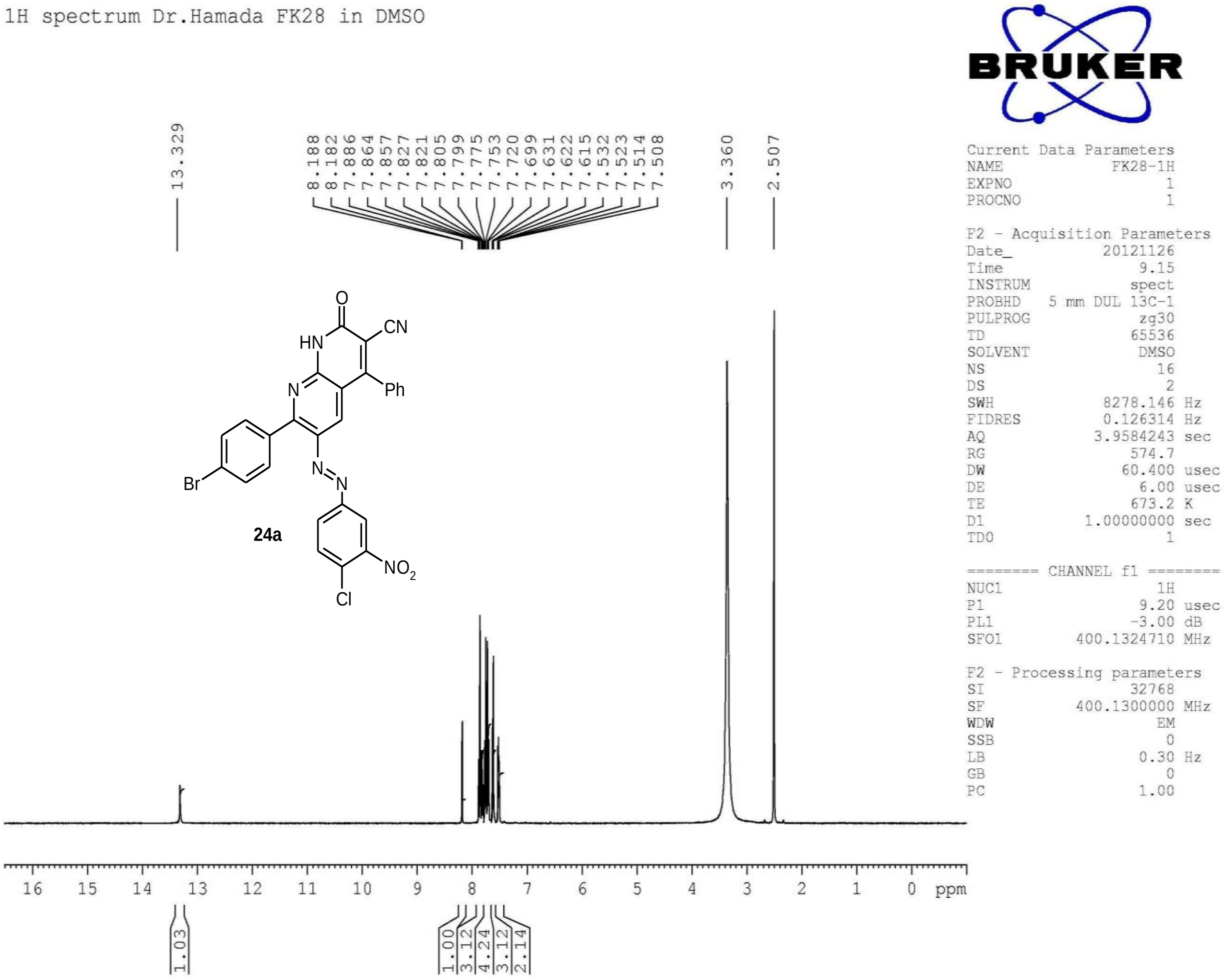

Figure S17. ^{1}H-NMR expansion of compound **24a**.

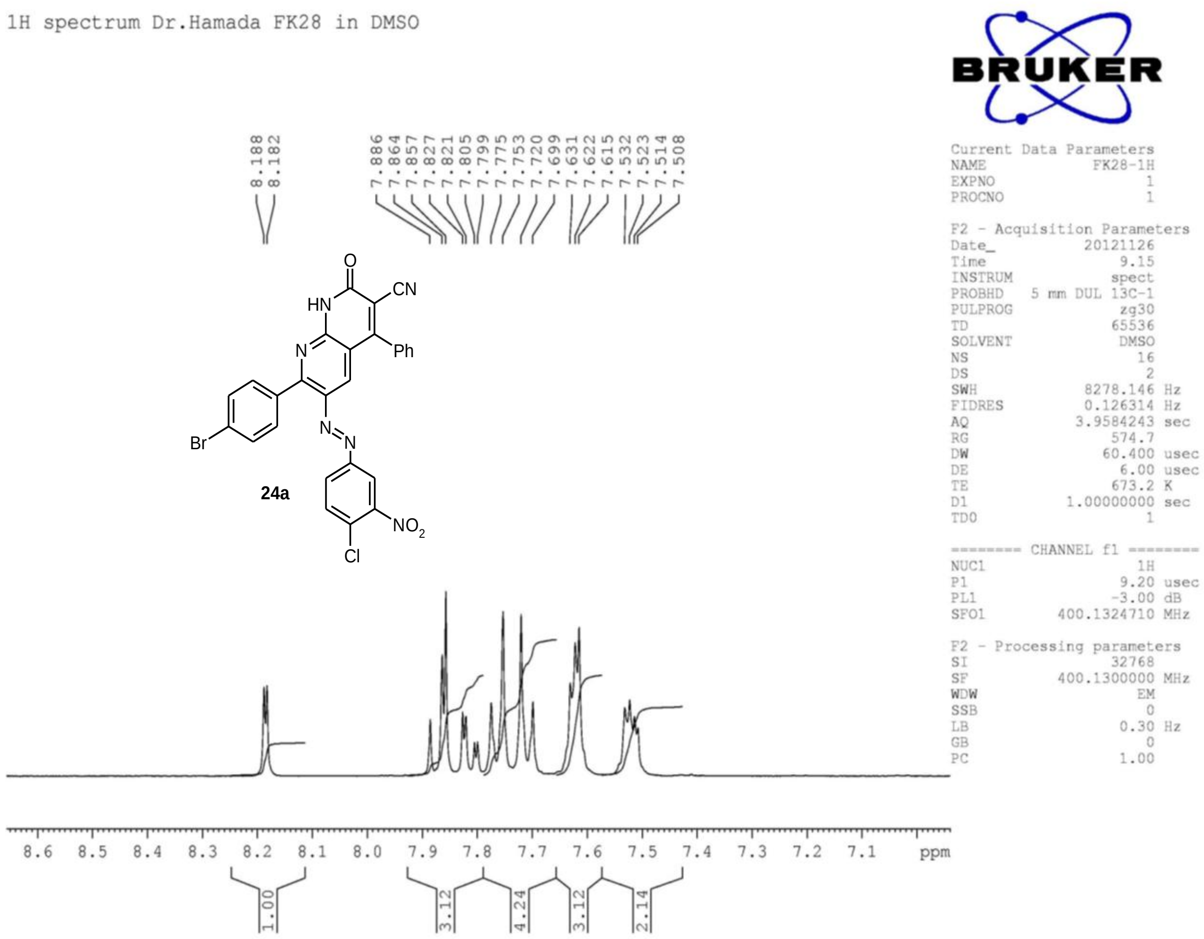

Figure S18. ^{13}C-NMR spectra of compound **24a**.

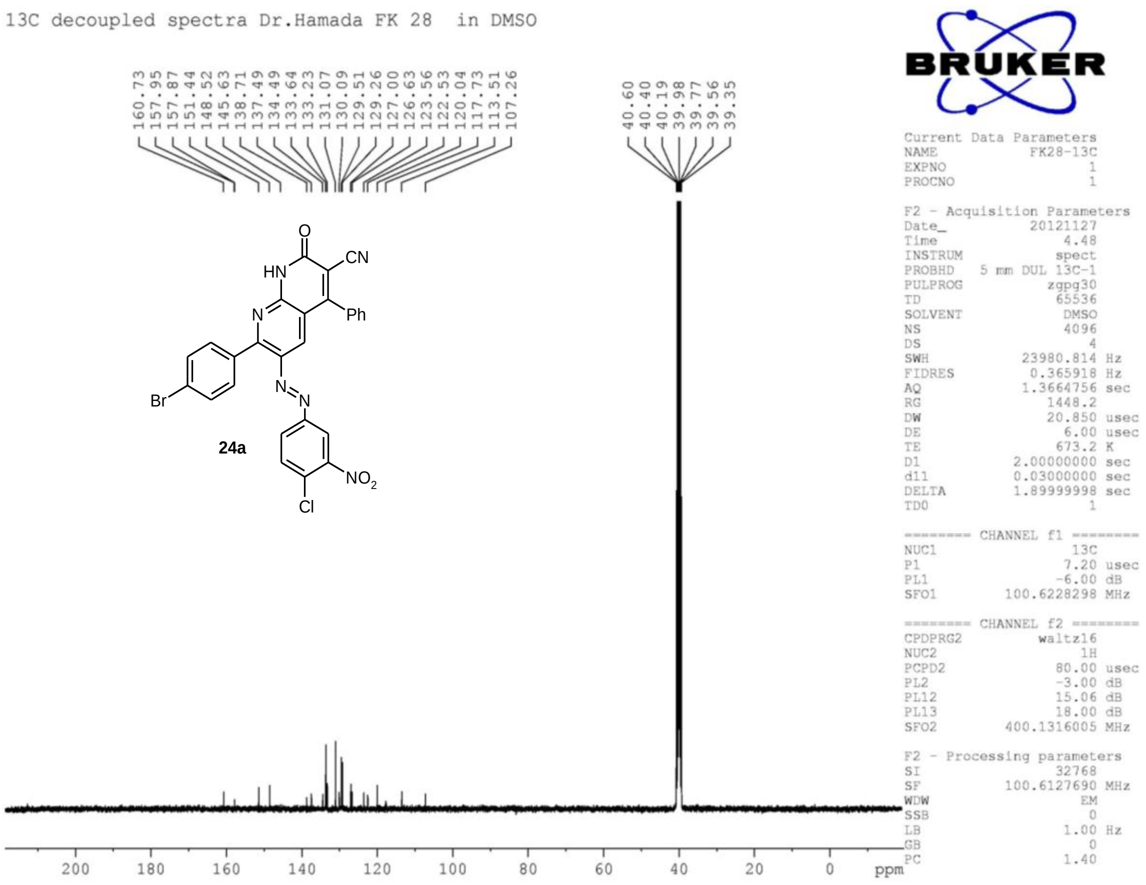

Figure S19. ^{1}H-NMR spectra of compound **24b**.

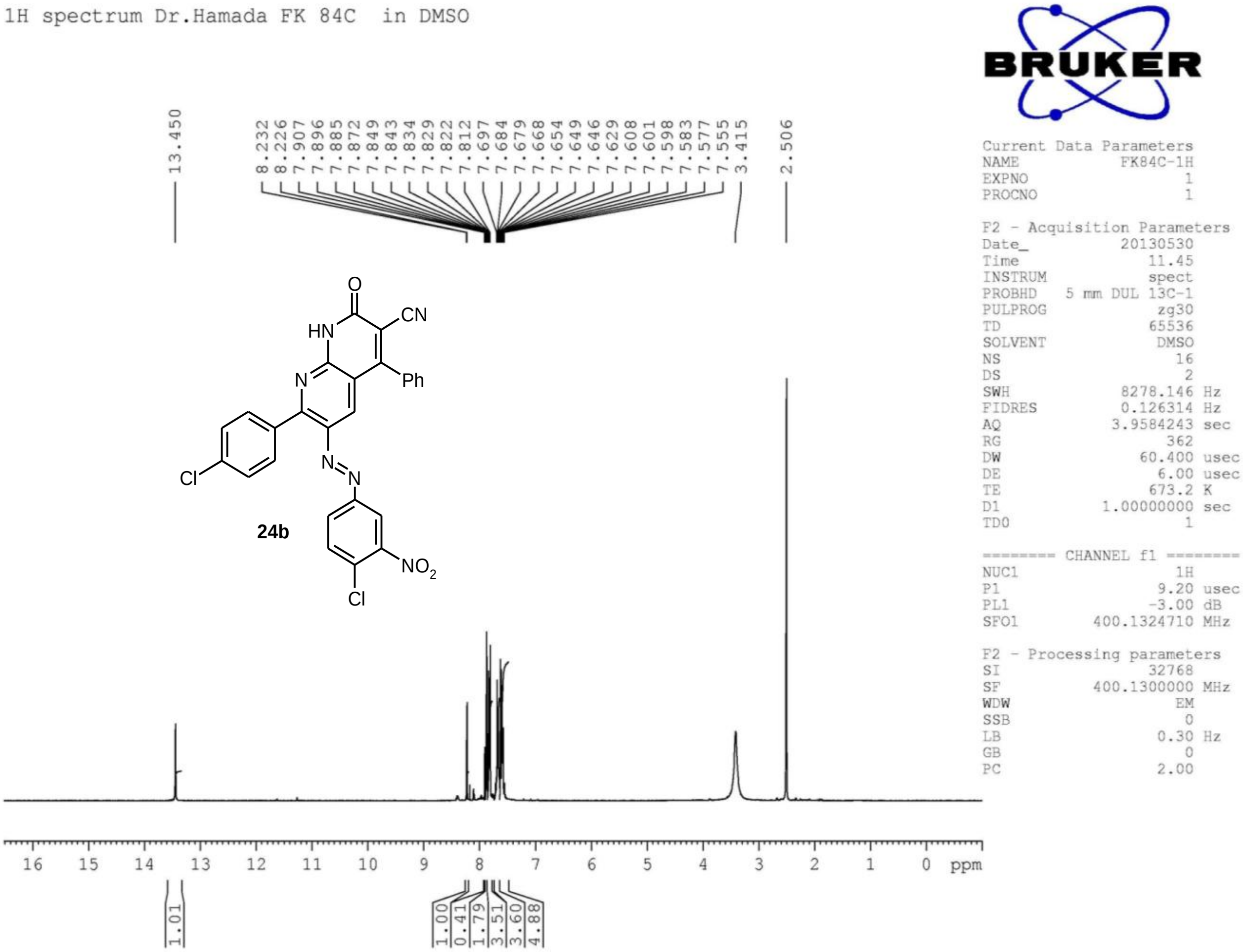

Figure S20. ^{1}H-NMR expansion of compound **24b**.

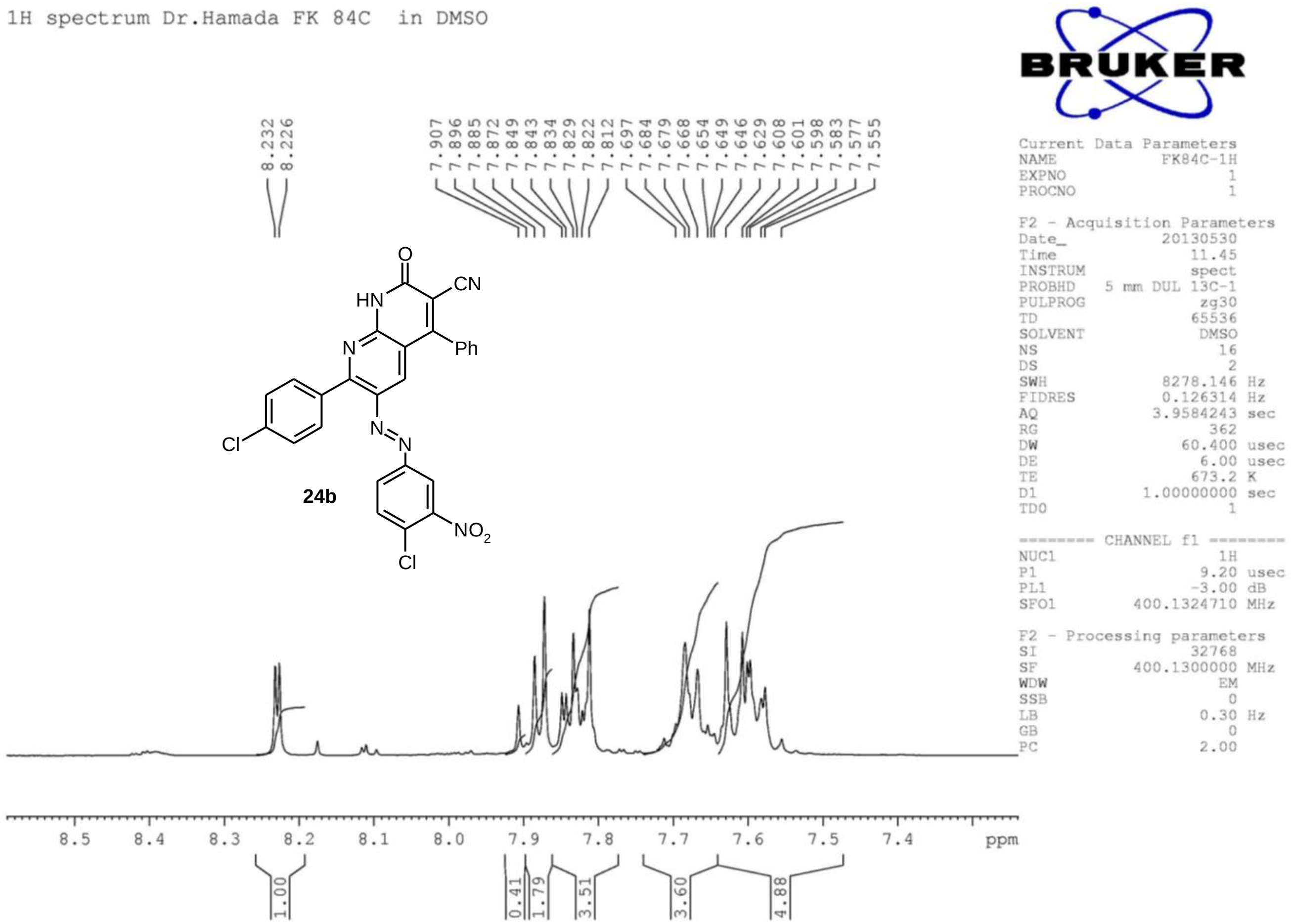

Figure S21. ^{13}C-NMR spectra of compound **24b**.

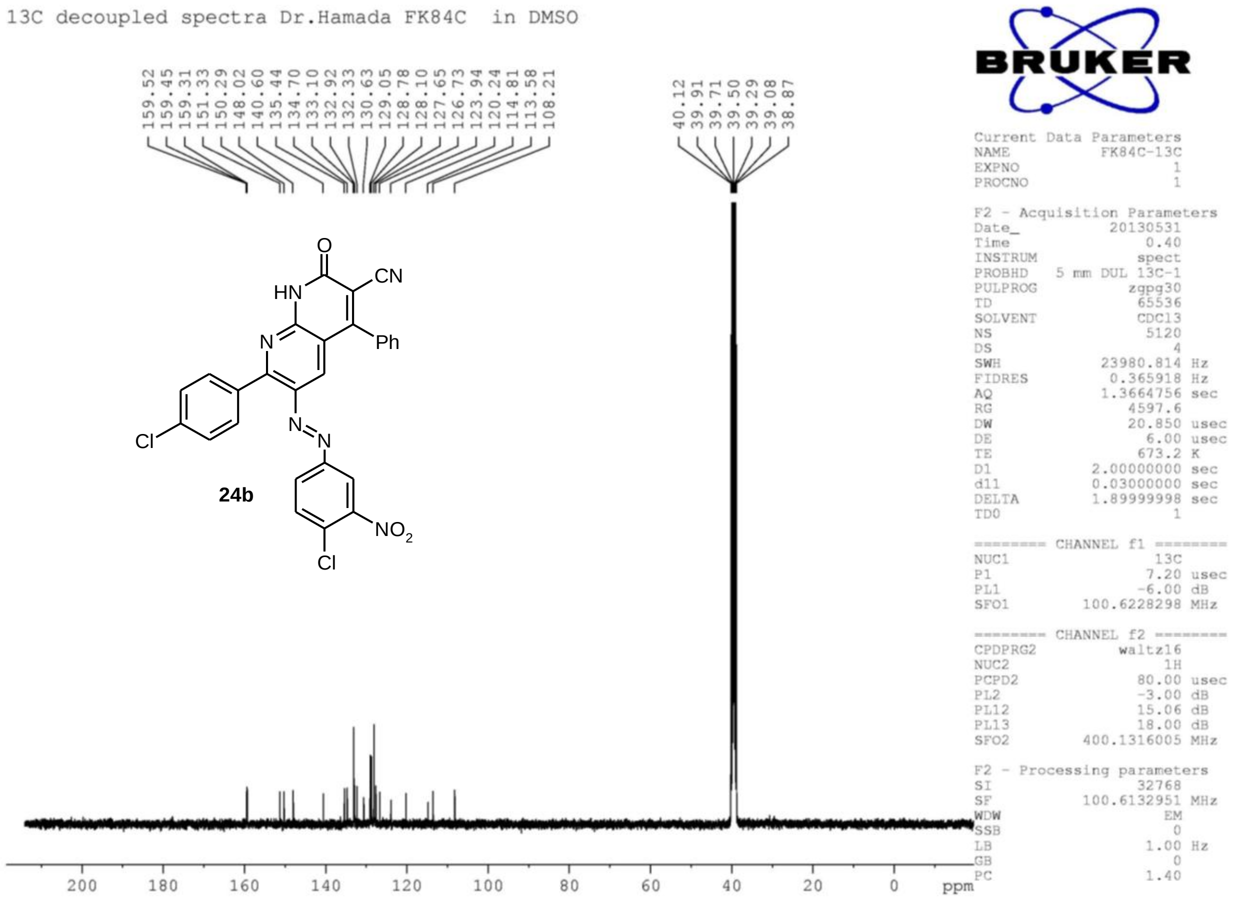

Supplement: Supplementary file 1 [file molecules-19-02637-s001.pdf]
